# SUV39H1 maintains cancer stem cell chromatin state and properties in glioblastoma

**DOI:** 10.1172/jci.insight.186344

**Published:** 2025-03-10

**Authors:** Chunying Li, Qiqi Xie, Sugata Ghosh, Bihui Cao, Yuanning Du, Giau V. Vo, Timothy Y. Huang, Charles Spruck, Richard L. Carpenter, Y. Alan Wang, Q. Richard Lu, Kenneth P. Nephew, Jia Shen

**Affiliations:** 1 Medical Sciences Program, and; 2 Cell, Molecular, and Cancer Biology Graduate Program, Indiana University School of Medicine, Bloomington, Indiana, USA.; 3 Degenerative Diseases Program, and; 4 Cancer Genome and Epigenetics Program, Sanford Burnham Prebys Medical Discovery Institute, La Jolla, California, USA.; 5 Department of Biochemistry and Molecular Biology, Indiana University School of Medicine, Indianapolis, Indiana, USA.; 6 Indiana University Melvin and Bren Simon Comprehensive Cancer Center, Indianapolis, Indiana, USA.; 7 Brown Center for Immunotherapy and Department of Medicine, Indiana University School of Medicine, Indianapolis, Indiana, USA.; 8 Brain Tumor Center, Division of Experimental Hematology and Cancer Biology, Cincinnati Children’s Hospital Medical Center, Cincinnati, Ohio, USA.; 9 Department of Pediatrics, University of Cincinnati, College of Medicine, Cincinnati, Ohio, USA.; 10 Department of Anatomy, Cell Biology and Physiology, and; 11 Department of Medical and Molecular Genetics, Indiana University School of Medicine, Indianapolis, Indiana, USA.

**Keywords:** Cell biology, Oncology, Stem cells, Cancer

## Abstract

Glioblastoma (GBM) is the most lethal brain cancer, with GBM stem cells (GSCs) driving therapeutic resistance and recurrence. Targeting GSCs offers a promising strategy for preventing tumor relapse and improving outcomes. We identify SUV39H1, a histone-3, lysine-9 methyltransferase, as critical for GSC maintenance and GBM progression. SUV39H1 is upregulated in GBM compared with normal brain tissues, with single-cell RNA-seq showing its expression predominantly in GSCs due to super-enhancer–mediated activation. Knockdown of SUV39H1 in GSCs impaired their proliferation and stemness. Whole-cell RNA-seq analysis revealed that SUV39H1 regulates G_2_/M cell cycle progression, stem cell maintenance, and cell death pathways in GSCs. By integrating the RNA-seq data with ATAC-seq data, we further demonstrated that knockdown of SUV39H1 altered chromatin accessibility in key genes associated with these pathways. Chaetocin, an SUV39H1 inhibitor, mimics the effects of SUV39H1 knockdown, reducing GSC stemness and sensitizing cells to temozolomide, a standard GBM chemotherapy. In a patient-derived xenograft model, targeting SUV39H1 inhibits GSC-driven tumor growth. Clinically, high SUV39H1 expression correlates with poor glioma prognosis, supporting its relevance as a therapeutic target. This study identifies SUV39H1 as a crucial regulator of GSC maintenance and a promising therapeutic target to improve GBM treatment and patient outcomes.

## Abstract 2

SUV39H1 maintains glioblastoma stem cells

## Introduction

Glioblastoma (GBM) is the most common primary malignant brain tumor in adults, known for its aggressiveness and lethality (1). Despite standard treatment that includes a combination of surgery, radiotherapy, and/or temozolomide (TMZ), the prognosis for patients with GBM remains poor, with a median survival of less than 16 months (2, 3). There is an urgent need for more effective treatments to improve patient survival.

The resistance of GBM to standard treatment is partly attributed to intratumoral heterogeneity driven by GBM stem cells (GSCs), which possess self-renewal and differentiation properties, and exhibit strong tumorigenic potential (4–6). GSCs can differentiate into various cell types within GBM, such as endothelial cells, pericytes, and non-stem GBM cells (NSGCs). As GSCs can initiate and propagate tumors, resist standard therapies including TMZ, repopulate tumors after treatment, and contribute to disease relapse, eliminating GSCs could overcome chemoresistance and represent an effective therapeutic strategy in GBM.

Epigenetic modifications, including DNA methylation and histone modifications, profoundly impact gene expression and cellular behavior in normal and cancer cells, including stem cells (7, 8). Targeting epigenetic regulators in GSCs has the potential to reverse aberrant gene expression patterns, disrupt stemness, and sensitize GSCs to existing therapies, offering new avenues for effective GBM treatment. Previous studies have identified several epigenetic regulators involved in maintaining GSC stemness and therapeutic resistance, including PRMT6 (9), PRC2 (10, 11), KDM2B (12), DNMT1 (13), HDACs (14, 15), and HELLS (16). SUV39H1 is a key epigenetic regulator and histone methyltransferase responsible for trimethylating histone H3 at lysine 9 (H3K9me3) (17, 18), which promotes heterochromatin formation and transcriptional repression. However, the specific roles of SUV39H1 in GSCs and GBM remain to be determined.

To address this gap, we employed an integrative approach, combining in vitro and in vivo experiments with bioinformatics analyses, to investigate the functional importance of SUV39H1 in GSCs and GBM progression.

## Results

### SUV39H1 is upregulated in GBM.

**Figure 1:**
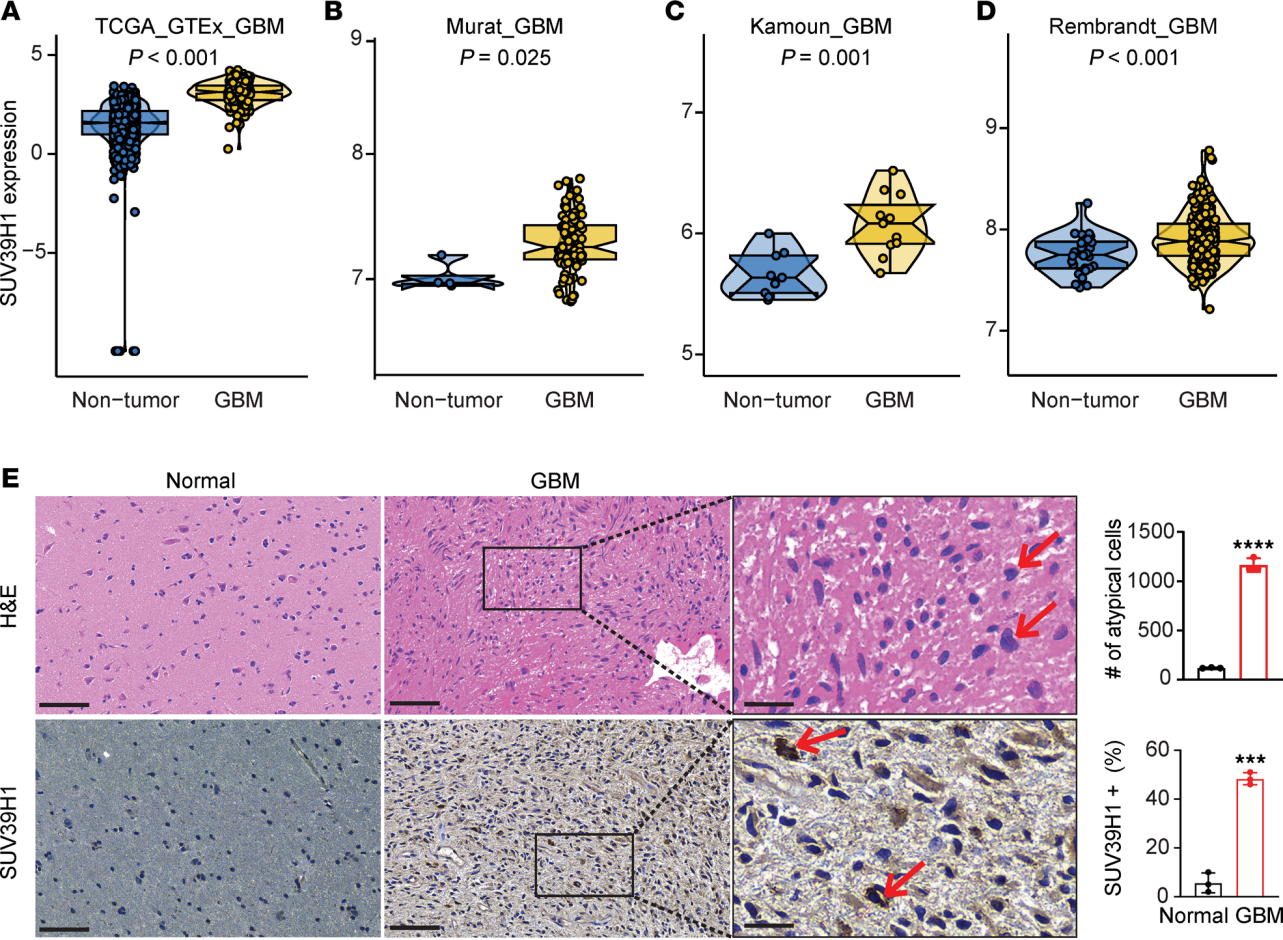
SUV39H1 is upregulated in GBM. (**A**–**D**) Violin plots showing SUV39H1 expression in nontumor and GBM tissues across 4 datasets: TCGA_GTEx_GBM (**A**), Murat_GBM (**B**), Kamoun_GBM (**C**), and Rembrandt_GBM (**D**). (**E**) H&E staining (top row) and IHC staining of SUV39H1 (bottom row) in normal (*n* = 4) and GBM (*n* = 9) tissues. Quantification of the number of atypical cells and the percentage of SUV39H1-positive cells is shown on the right. Scale bars: 100 μm (left and middle panels) and 30 μm (high-magnification insets, right panel). Data represent mean ± SD. ***P < 0.001, ****P < 0.0001 by unpaired, 2-tailed *t* test.

We first investigated the expression of SUV39H1 in GBM using RNA-seq data from GBM datasets (The Cancer Genome Atlas [TCGA], Murat, Kamoun, and Rembrandt). Compared with nontumor brain tissues, SUV39H1 expression was significantly increased in GBM tissues (*P* < 0.05; Figure 1, A–D). Overexpression of SUV39H1 in GBM tissues was confirmed by immunohistochemistry (IHC) (*P* < 0.001; Figure 1E). These findings demonstrate that SUV39H1 is upregulated in GBM compared with normal brain tissues.

### SUV39H1 is preferentially expressed in GSCs.

**Figure 2:**
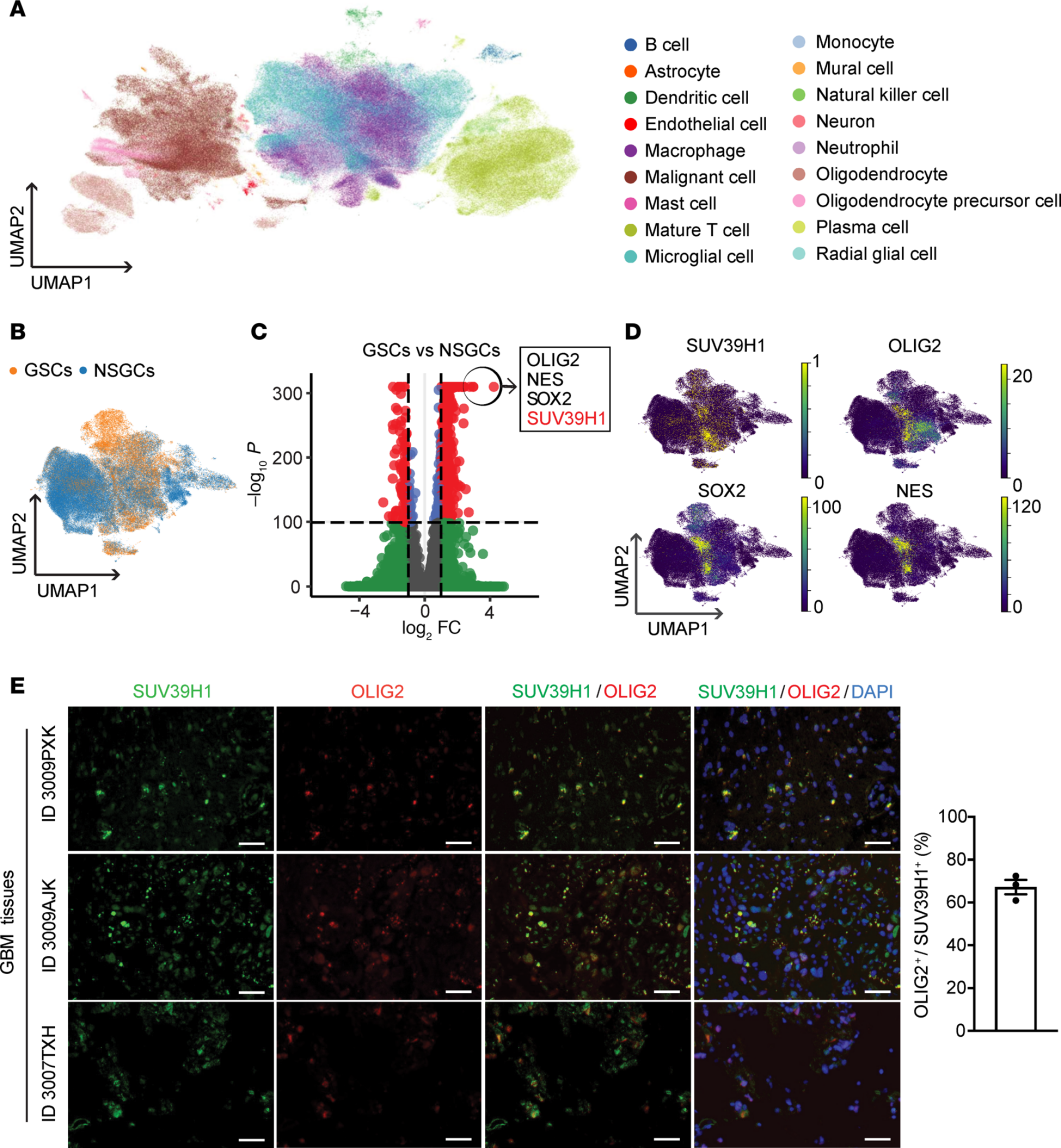
Expression patterns of SUV39H1 in GBM. (**A**) The UMAP clustering of single-cell RNA-seq data from GBM tumors sourced from the CELLxGENE database reveals various cell types. (**B**) UMAP plot distinguishing GSCs (orange) from non-stem GBM cells (NSGCs, blue). (**C**) Volcano plot displaying DEGs between GSCs and NSGCs. Genes with significant expression changes (|log_2_ fold change| > 2 and adjusted *P* value < 0.05) are shown as red dots, with the top genes (*OLIG2*, *NES*, *SOX2*, and *SUV39H1*) annotated in the box. (**D**) UMAP plots showing the expression patterns of the indicated genes in GSCs and NSGCs. The color intensity represents the normalized expression level. (**E**) Representative images (left panel) and quantification (right panel) of immunofluorescent staining showing colocalization of SUV39H1 (green) and OLIG2 (red) in GBM tissues (*n* = 3). Scale bars: 50 μm.

To examine the expression pattern of SUV39H1 in GBM, single-cell RNA-seq analysis was performed. Uniform manifold approximation and projection (UMAP) clustering identified distinct cell types in GBM (Figure 2A and Supplemental Figure 1; supplemental material available online with this article; https://doi.org/10.1172/jci.insight.186344DS1). We used stemness markers, including OLIG2, NES, and SOX2, to further analyze the malignant cells and distinguish GSCs from NSGCs (Figure 2B). The data revealed that SUV39H1 was preferentially overexpressed in GSCs (Figure 2, C and D). Immunofluorescent staining confirmed the colocalization of SUV39H1 with OLIG2 and SOX2 in GBM patient tissues (Figure 2E and Supplemental Figure 2).

**Figure 3:**
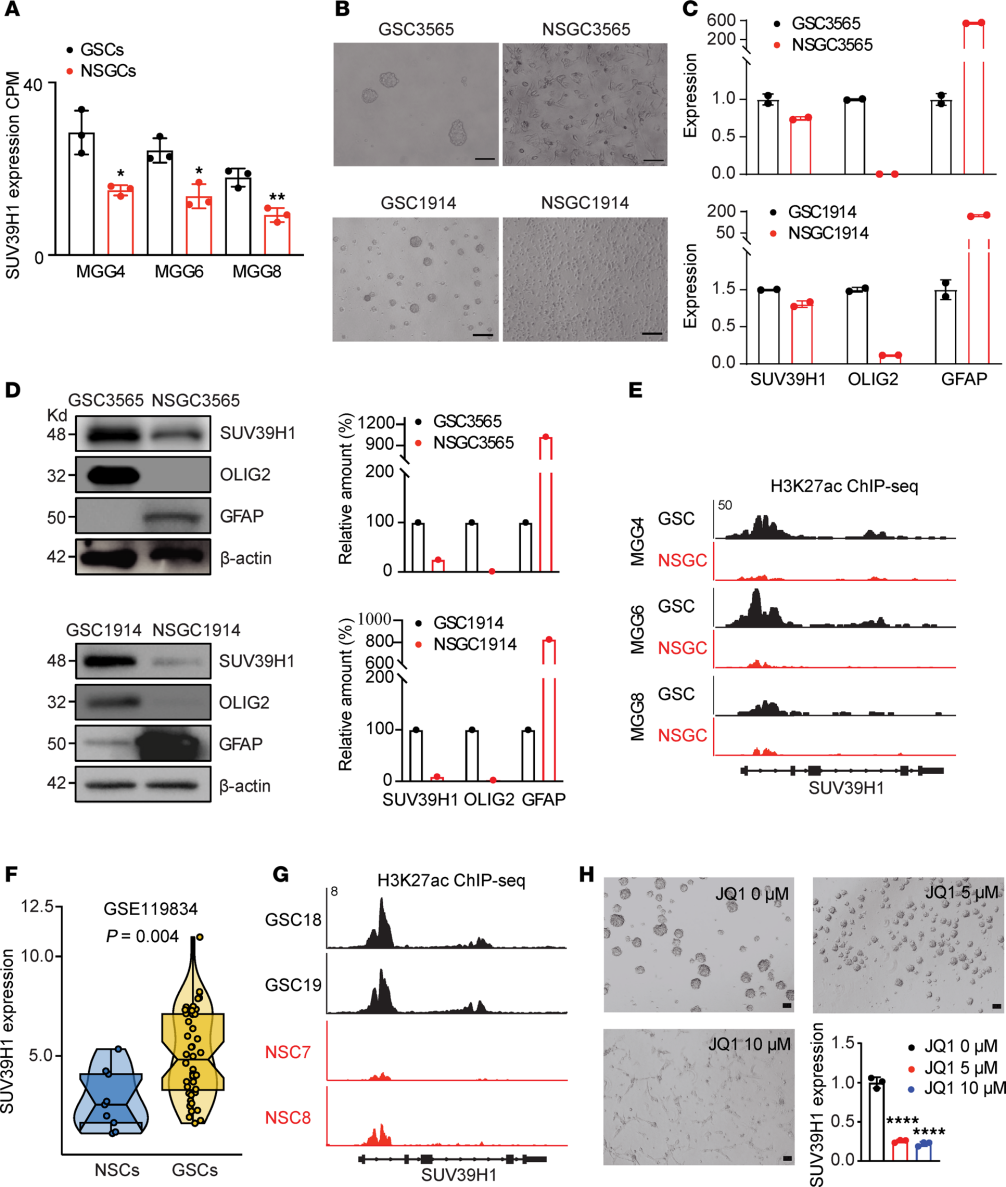
SUV39H1 expression in GSCs, NSGCs, and normal brain cells. (**A**) Analysis of RNA-seq data (GSE54791) for SUV39H1 expression in multiple GSC and NSGC pairs (MCG4, MCG6, MCG8). CPM, counts per million. (**B**–**D**) Representative images (**B**), qPCR data (**C**), and Western blotting data and quantification (**D**) for GSC3565 and GSC1914 differentiation by serum induction. OLIG2 is a GSC marker, and GFAP is a differentiated NSGC marker. (**E**) H3K27ac ChIP-seq data showing the *SUV39H1* locus in indicated GSCs and NSGCs. (**F**) Violin plot showing SUV39H1 expression levels in GSCs compared to normal neural stem cells (NSCs). *P* value was calculated using an unpaired, 2-tailed *t* test. (**G**) H3K27ac ChIP-seq signal tracks showing the *SUV39H1* locus in indicated GSCs and NSCs. (**H**) Representative images and qPCR data for GSC3565 cells treated with JQ1 for 48 hours. Scale bars: 100 μm. Data represent mean ± SD. *P < 0.05; **P < 0.01; ****P < 0.0001 by unpaired, 2-tailed *t* test.

Analysis of RNA-seq data for 3 pairs of GSCs and their matched differentiated NSGCs (NCBI Gene Expression Omnibus [GEO] GSE54791) revealed higher SUV39H1 expression in GSCs (*P* < 0.01; Figure 3A). Using GSC3565 and GSC1914 models for differentiated and undifferentiated cell states (Figure 3B), we found that SUV39H1 levels decreased during serum-induced differentiation, accompanied by downregulation of OLIG2 and upregulation of GFAP, a differentiated-NSGC marker (quantitative PCR [qPCR], Figure 3C; Western blot, Figure 3D).

ChIP-seq data of H3K27ac, a histone modification linked to active super-enhancers that boost gene transcription, revealed enriched peaks at the *SUV39H1* gene in GSCs compared with differentiated NSGCs, indicating enhanced regulatory activity of SUV39H1 expression in GSCs (Figure 3E). Additionally, RNA-seq analysis of 44 GSC models and 9 normal brain cell lines (GSE119834) demonstrated preferential SUV39H1 expression in GSCs (*P* = 0.004; Figure 3F). Consistently, ChIP-seq signal tracks for H3K27ac showed increased super-enhancer marks at the *SUV39H1* gene in GSCs versus normal brain cells (Figure 3G). To confirm the role of GSC-specific super-enhancer regulation of SUV39H1 expression, we treated GSCs with JQ1, a bromodomain-containing 4 (BRD4) inhibitor known to disrupt super-enhancer function (19). This treatment led to a dose-dependent reduction in SUV39H1 mRNA levels (Figure 3H), demonstrating that SUV39H1 expression in GSCs is indeed super-enhancer driven. We further examined available ChIP-seq data for OLIG2 and SOX2 in GSCs (20) and identified binding peaks for both transcription factors at the *SUV39H1* locus, overlapping partially with H3K27ac peaks (Supplemental Figure 3A), suggesting their potential role in regulating SUV39H1 expression. Knockdown (KD) studies showed that silencing SOX2 led to a reduction in SUV39H1 mRNA expression (Supplemental Figure 3, B and C), and OLIG2 KD similarly decreased SUV39H1 mRNA levels (Supplemental Figure 3, D and E). Furthermore, SUV39H1 expression was positively correlated with SOX2 and OLIG2 expression in GBM samples from the TCGA (Supplemental Figure 3F) and Chinese Glioma Genome Atlas (CGGA) databases (Supplemental Figure 3G). Together, these findings indicate that SOX2 and OLIG2 may promote SUV39H1 expression through their interaction with super-enhancers. Further studies are needed to clarify the detailed mechanism.

### SUV39H1 is required for GSC maintenance.

**Figure 4:**
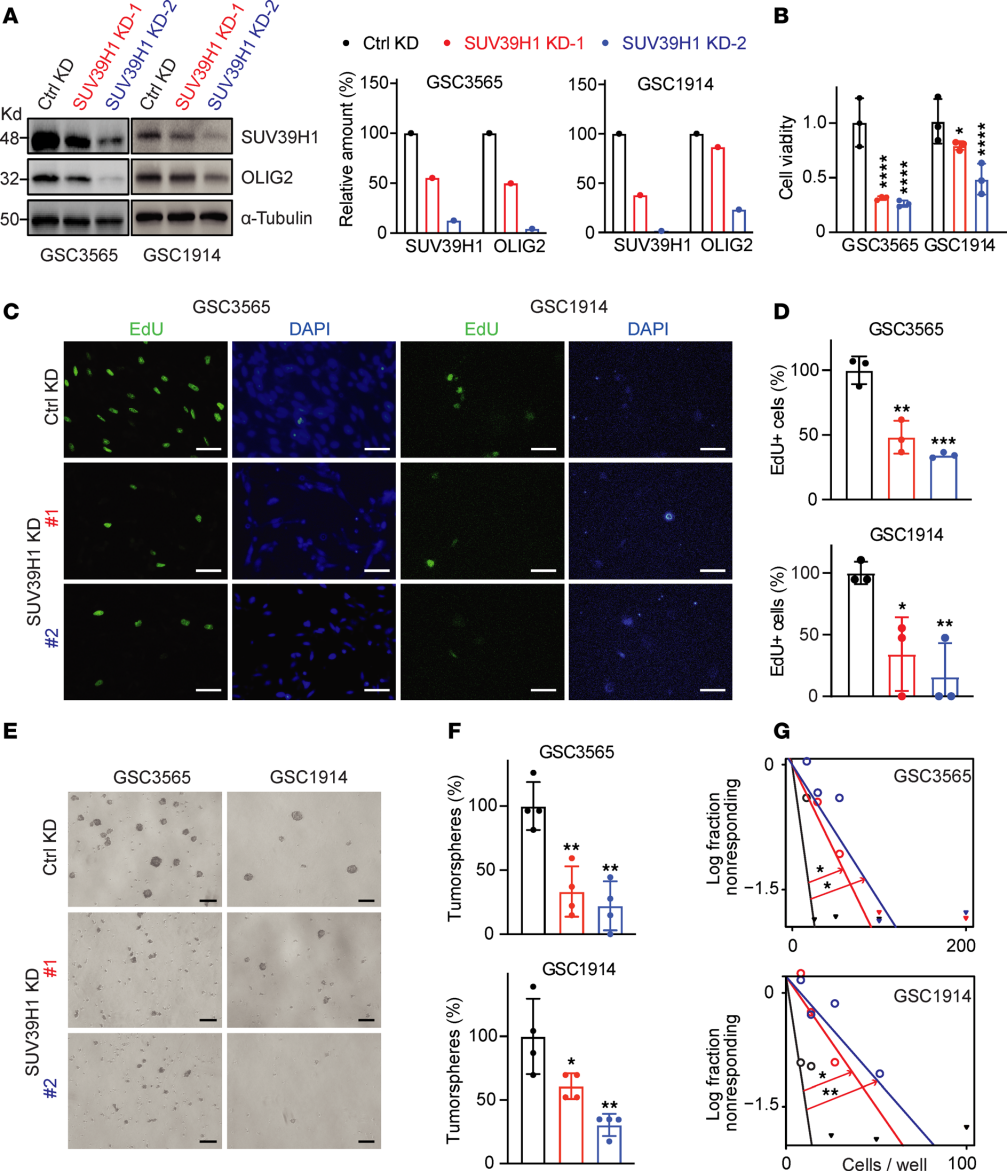
SUV39H1 regulates GSC proliferation and stemness. (**A**) Western blot data (left panel) and quantification (right panel) showing the levels of SUV39H1 and OLIG2 in control (Ctrl) and SUV39H1-KD GSCs. (**B**) Cell viability analysis for GSC3565 (day 5) and GSC1914 (day 8) following gene KD. Two-way ANOVA and Dunnett’s multiple-comparison test. (**C** and **D**) Representative images (**C**) and quantification (**D**) of EdU incorporation assay in GSCs. Unpaired, 2-tailed *t* test. Scale bars: 50 μm. (**E** and **F**) Representative images (**E**) and quantification (**F**) of tumorsphere formation in GSCs after 72 hours of gene KD. Unpaired, 2-tailed *t* test. Scale bars: 100 μm. (**G**) Limiting dilution assay demonstrating the self-renewal capacity of GSCs with various cell numbers after 13 days of gene KD. Data points represent the log fraction of wells without spheres plotted against the number of cells plated per well. Pairwise test. Data represent mean ± SD. *P < 0.05; **P < 0.01; ***P < 0.001; ****P < 0.0001.

SUV39H1 upregulation in GSCs suggests a potential dependency on this enzyme. To investigate the functional roles of SUV39H1 in GSCs, we first knocked down SUV39H1 in GSC3565 and GSC1914 cells using 2 different shRNAs and confirmed KD efficiency by Western blotting (Figure 4A). SUV39H1 KD led to decreased GSC proliferation (Figure 4B), which was further validated by a 5-ethynyl-2′-deoxyuridine (EdU) incorporation assay showing slower DNA replication in GSCs with SUV39H1 KD (Figure 4, C and D). In NSGCs differentiated from GSCs (Supplemental Figure 4, A–C) and in U118 (Supplemental Figure 4, D–F), a human GBM cell line, SUV39H1 KD also reduced proliferation and survival, suggesting that both GSCs and NSGCs may depend on SUV39H1 for these functions. Our prior research demonstrated that targeting SUV39H1 had minimal impact on nonneoplastic cells, such as astrocytes and human mammary epithelial cells, in contrast with its significant effects in GSCs (21). Consistent with this, recent research also revealed that SUV39H1 KD did not affect the proliferation of astrocytes or human neural stem cells (22). Furthermore, SUV39H1 KD impaired the self-renewal ability of GSCs. Tumorsphere formation assay showed a significant reduction in the number of tumorspheres formed by SUV39H1-KD cells (*P* < 0.05; Figure 4, E and F). Extreme limiting dilution assay (ELDA) quantitatively demonstrated a decreased stem cell frequency in SUV39H1-KD cells (*P* < 0.05; Figure 4G). Notably, in GSC3565 and GSC1914 cells, shSUV39H1-2 achieved higher KD efficiency than shSUV39H1-1, correlating with greater reductions in proliferation (Figure 4B) and stemness (Figure 4, F and G). This dose-dependent relationship underscores the critical role of SUV39H1 in these processes. In addition to using GSC3565 (undefined GBM subtype) and GSC1914 (classical subtype), we assessed SUV39H1 KD in GSCs from other GBM subtypes, including GSC23 (proneural subtype) and GSC839 (mesenchymal subtype). Consistent with previous findings, SUV39H1 KD reduced tumorsphere growth and viability across these subtypes (Supplemental Figure 4, G–L).

**Figure 5:**
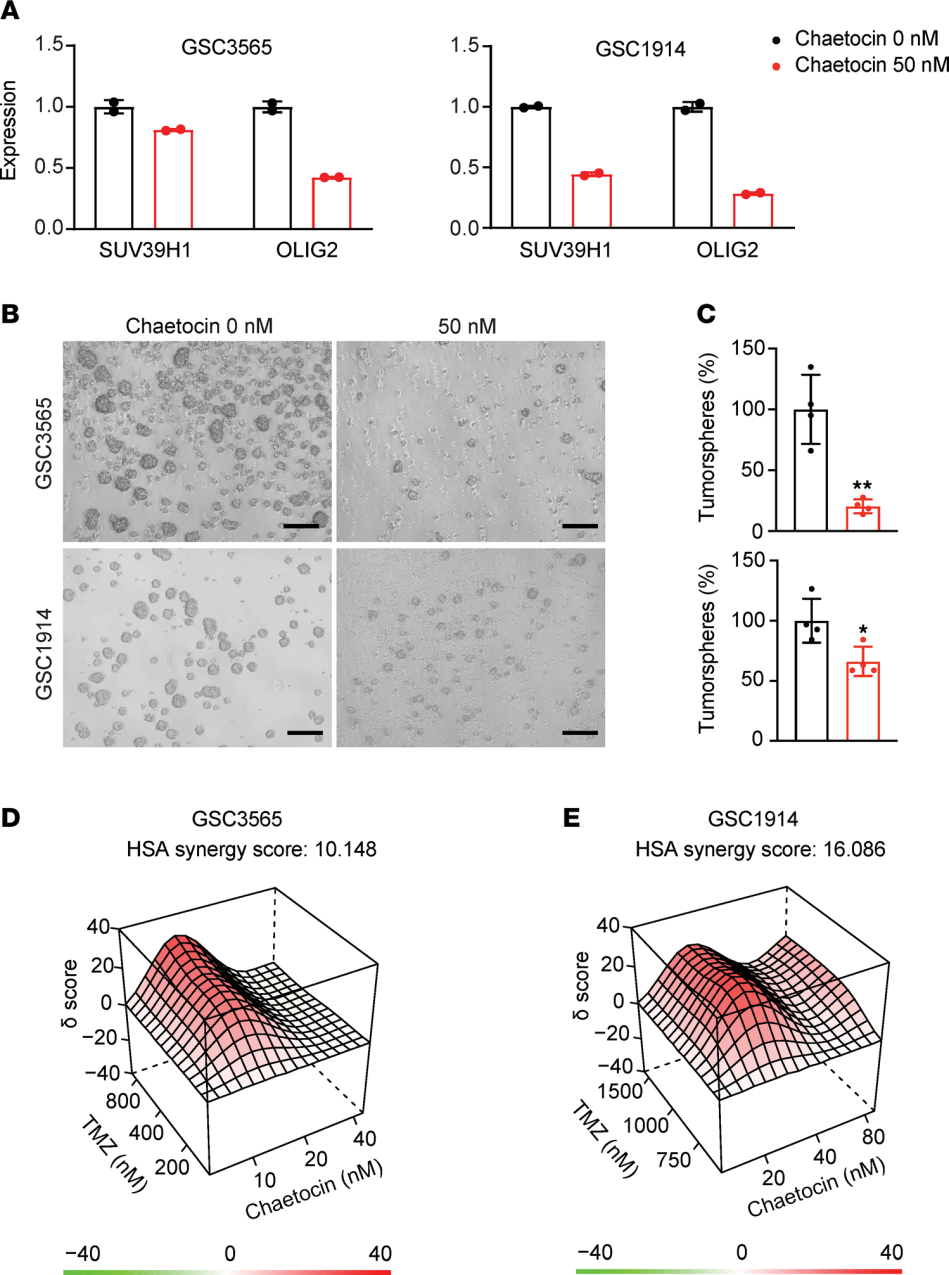
Chaetocin treatment disrupts GSCs and synergizes with TMZ. (**A**) qPCR analysis of SUV39H1 and OLIG2 expression in GSCs treated with 0 nM or 50 nM chaetocin. (**B** and **C**) Representative images (**B**) and quantification (**C**) of tumorsphere formation in GSCs after 48 hours of chaetocin treatment. Unpaired, 2-tailed *t* test. Scale bars: 100 μm. (**D** and **E**) 3D synergy score plots showing the synergistic effect of chaetocin and TMZ on killing GSC3565 (**D**) and GSC1914 (**E**). The highest single agent (HSA) synergy score assesses the combination’s efficacy relative to the most effective single agent involved in the combination. An HSA score of greater than 10 is considered indicative of synergy. Data represent mean ± SD. *P < 0.05, **P < 0.01.

To further examine the effect of inhibiting SUV39H1, GSCs were treated with chaetocin, an inhibitor of SUV39H1 (22, 23). qPCR analysis demonstrated decreased expression of SUV39H1 and OLIG2 in GSCs treated with chaetocin compared with untreated controls (Figure 5A). Tumorsphere formation assay revealed a significant reduction in the number of tumorspheres formed in chaetocin-treated GSCs (*P* < 0.05; Figure 5, B and C). Notably, chaetocin treatment sensitized both GSC3565 and GSC1914 cells to the GBM chemotherapy drug TMZ, with synergy scores of 10.148 and 16.086, respectively (Figure 5, D and E). These findings demonstrate that SUV39H1 is essential for GSC maintenance.

### SUV39H1 regulates cell cycle, stemness, and cell death pathways in GSCs.

**Figure 6:**
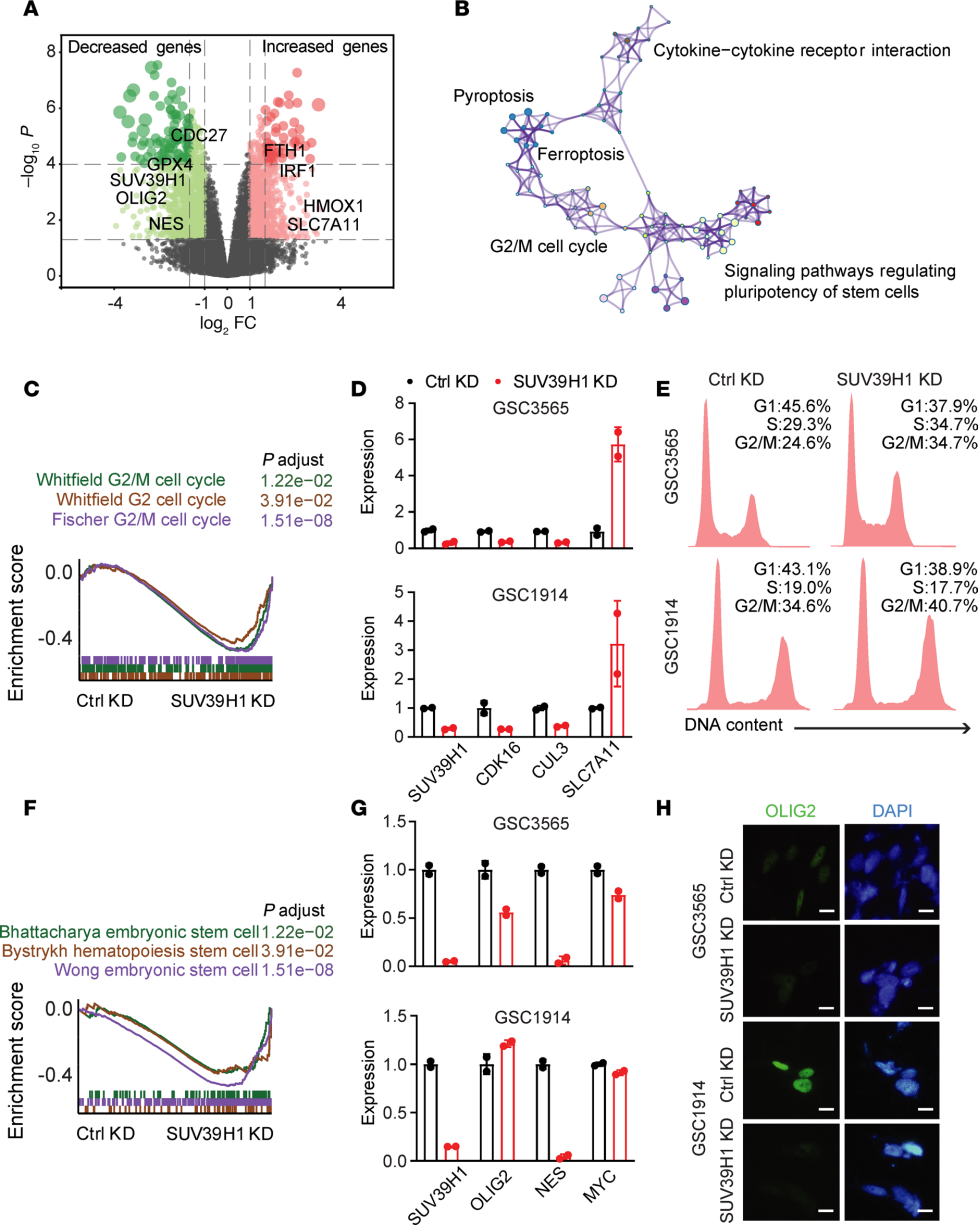
Signaling pathways regulated by SUV39H1 in GSCs. (**A**) Volcano plot illustrating DEGs in SUV39H1-KD versus control KD GSCs, with key genes highlighted. (**B**) Enrichment map visualizing the significant pathways affected by SUV39H1 KD. (**C**) GSEA plot showing enrichment of the G_2_/M cell cycle–related pathways in GSCs with SUV39H1 KD. *P*.adjust values indicate the significance of enrichment, which was assessed using the Kolmogorov-Smirnov test. Multiple hypothesis testing correction was applied using the Benjamini-Hochberg method to control the false discovery rate (FDR). (**D**) qPCR detection of G_2_/M cell cycle–related genes in GSC3565 and GSC1914 cells. (**E**) Flow cytometry data showing cell cycle change in GSCs with SUV39H1 KD. (**F**) GSEA plot showing enrichment of the stem cell–related pathways in SUV39H1-KD GSCs. (**G**) qPCR detection of stem cell–related genes in GSCs. (**H**) Immunofluorescent staining for OLIG2 (green) and DAPI (blue) in GSC3565 and GSC1914 cells. Scale bars: 50 μm.

To explore the molecular mechanisms by which SUV39H1 maintains GSCs, we performed RNA-seq analysis on GSC3565 and GSC1914 cells with SUV39H1 KD versus control KD (Figure 6A). Gene set enrichment analysis (GSEA) identified several enriched pathways associated with the differentially expressed genes (DEGs) (Figure 6B). SUV39H1-KD GSCs exhibited downregulation of G_2_/M cell cycle pathways (Figure 6C). Consistently, qPCR detection of G_2_/M cell cycle–related genes, including *CDK16*, *CDC27*, and *CUL3*, showed decreased expression upon SUV39H1 KD (Figure 6D). Flow cytometry analysis revealed an enrichment of the G_2_/M phase cell population in SUV39H1-KD GSCs (Figure 6E). A similar increase in the G_2_/M phase population was also observed in NSGCs differentiated from GSCs and in U118 cells with SUV39H1 KD (Supplemental Figure 5A). GSEA also identified decreased stem cell–related pathways in SUV39H1-KD cells (Figure 6F), which was validated by qPCR showing downregulation of stemness genes, including *OLIG2*, *NES*, and *MYC* (Figure 6G), and by immunofluorescence for OLIG2 (Figure 6H). Additionally, cell death pathways were upregulated, with GSEA indicating enrichment of autophagy, ferroptosis, and pyroptosis pathways (Supplemental Figure 5B), which were validated by qPCR for selected genes (Supplemental Figure 5C). These results demonstrate that SUV39H1 regulates cell cycle, stemness, and cell death pathways in GSCs.

### Targeting SUV39H1 alters chromatin accessibility.

**Figure 7:**
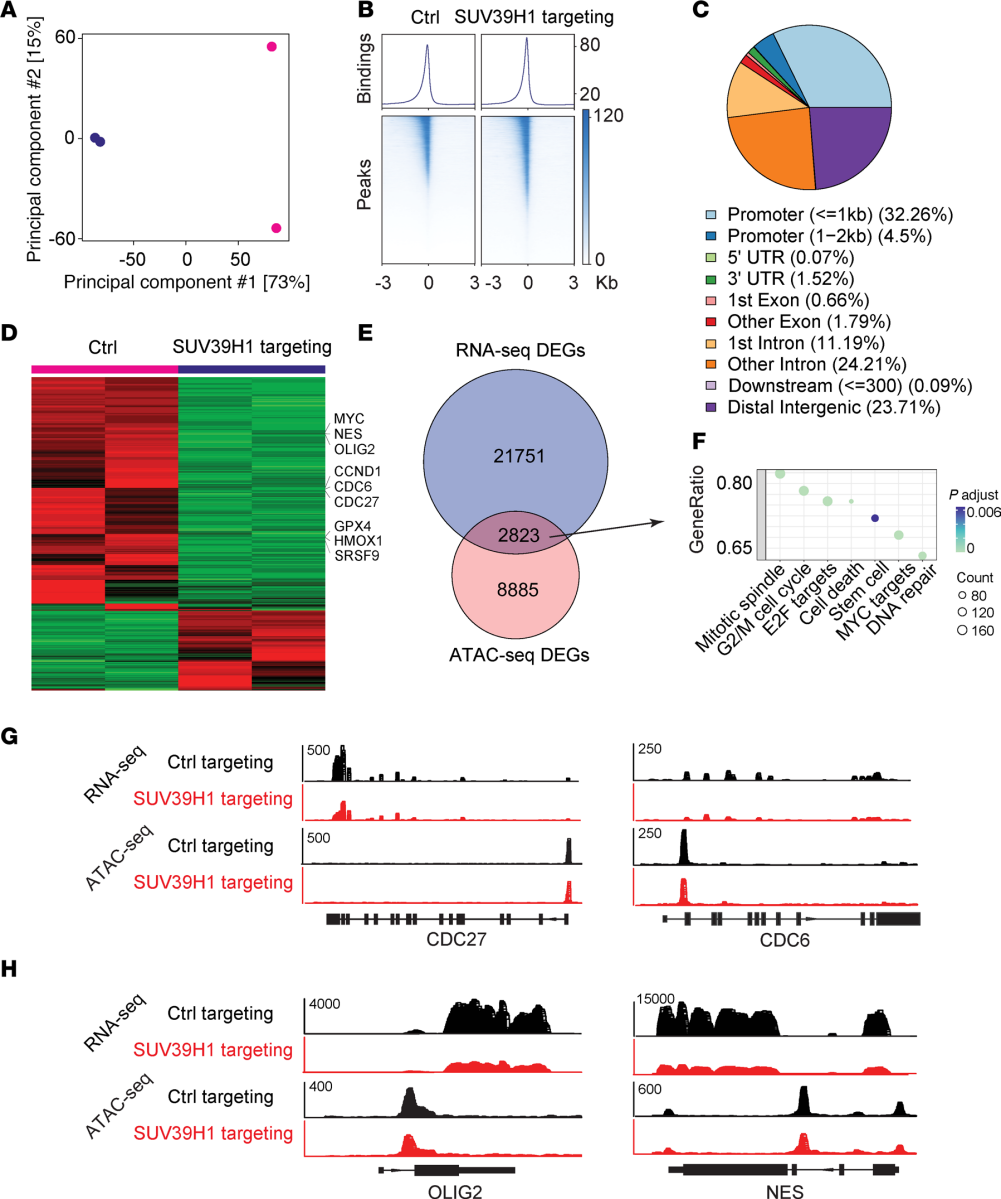
Targeting SUV39H1 alters chromatin accessibility in GSCs. (**A**) Principal component analysis (PCA) plot displaying the clustering of untreated (blue) and chaetocin-treated (pink) GSCs. (**B**) Aggregate plots (top) and heatmaps (bottom) of ATAC-seq signals at transcription start sites (TSS) in untreated and chaetocin-treated GSCs. (**C**) Pie chart illustrating the distribution of differentially accessible chromatin regions across various genomic features in response to chaetocin treatment. (**D**) Heatmap demonstrating the genes associated with altered chromatin accessibility upon SUV39H1 targeting. (**E**) Venn diagram showing the overlap of genes (*n* = 2823, 8.4%) between RNA-seq DEGs (blue) and genes associated with ATAC-seq differentially accessible regions (pink) after chaetocin treatment. (**F**) Bubble plot of GO enrichment analysis for the overlapping genes identified in **E**. (**G** and **H**) RNA-seq and ATAC-seq signals for indicated genes related to G_2_/M cell cycle (**G**) and stem cell maintenance (**H**).

To investigate how targeting SUV39H1 affects gene expression, we performed an assay for transposase-accessible chromatin followed by sequencing (ATAC-seq), revealing distinct chromatin accessibility profiles between untreated and chaetocin-treated GSCs (Figure 7, A and B). These differentially accessible chromatin regions were distributed across various genomic features (Figure 7C), and heatmap visualization highlighted a significant population of genes associated with these regions, which we named ATAC-seq differential genes (ATAC-seq-DGs) (Figure 7D). Integrating ATAC-seq-DGs with RNA-seq DEGs (Figure 6A) identified overlapping genes (*n* = 2823, 8.4%) (Figure 7E), with many linked to G_2_/M cell cycle and stem cell pathways (Figure 7F). For example, *CDC27* and *CDC6* G_2_/M cell cycle (Figure 7G) and *OLIG2* and *NES* stemness genes (Figure 7H) showed decreased chromatin accessibility and gene expression upon SUV39H1 targeting, as visualized in the ATAC-seq and RNA-seq signal tracks. These data suggest that targeting SUV39H1 reduces chromatin accessibility at these genes, resulting in downregulation of their expression, contributing to G_2_/M cell cycle arrest and disruption of stemness.

### Targeting SUV39H1 decreases GSC-driven GBM growth in mice.

**Figure 8:**
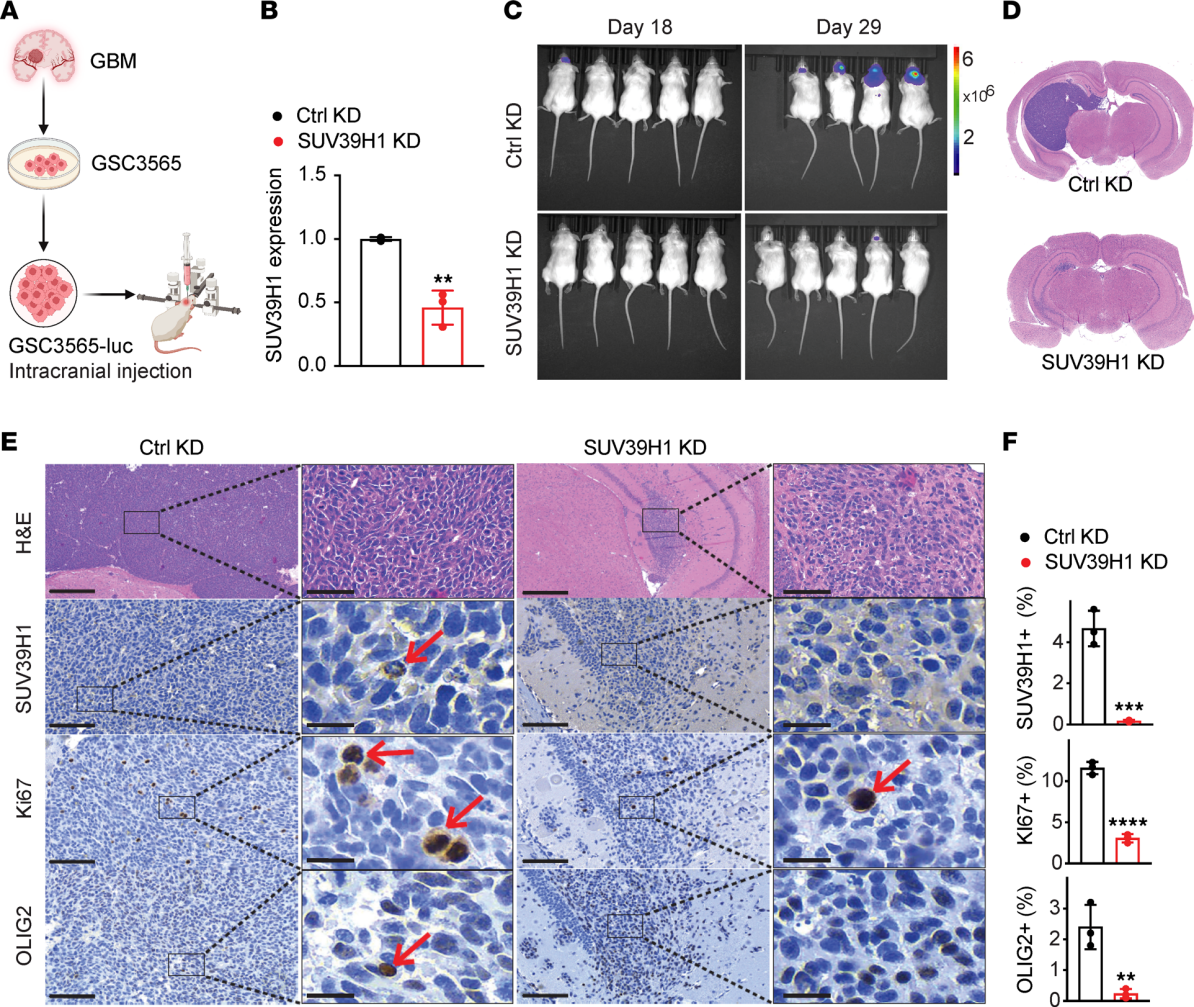
Targeting SUV39H1 decreases GSC-driven GBM growth in mice. (**A**) Schematic representation of the experimental design for intracranial injection of GSCs with control or SUV39H1 KD. (**B**) qPCR analysis of SUV39H1 expression in GSCs prior to injection. (**C**) Bioluminescence imaging of tumor-bearing mice on days 18 and 29 after implantation of GSCs. (**D**) Representative H&E-stained coronal brain sections of mice implanted with indicated GSCs on day 29. (**E** and **F**) Representative images (**E**) and quantification (**F**) of IHC staining for SUV39H1, Ki67, and OLIG2 in tumor sections from **D**. Scale bars: 100 μm (left panels) and 20 μm (high-magnification insets, right panels). Data represent mean ± SD. **P < 0.01, ***P < 0.001, ****P < 0.0001 by unpaired, 2-tailed *t* test.

The impact of targeting SUV39H1 on the in vivo tumor formation ability of GSCs was assessed using a xenograft mouse model. Control or SUV39H1-KD GSC3565 cells expressing luciferase were intracranially injected into the brains of immunodeficient mice (Figure 8, A and B). SUV39H1-KD GSC–derived tumors displayed significantly reduced growth in mice on day 29 (Figure 8, C and D). IHC analysis revealed decreased staining for SUV39H1, Ki67, and OLIG2 in SUV39H1-KD tumors compared with the controls (*P* < 0.001; Figure 8, E and F), demonstrating that targeting SUV39H1 can disrupt GSC proliferation and stemness, thereby inhibiting GBM growth in vivo.

### SUV39H1 serves as a therapeutic target and prognostic indicator in GBM.

**Figure 9:**
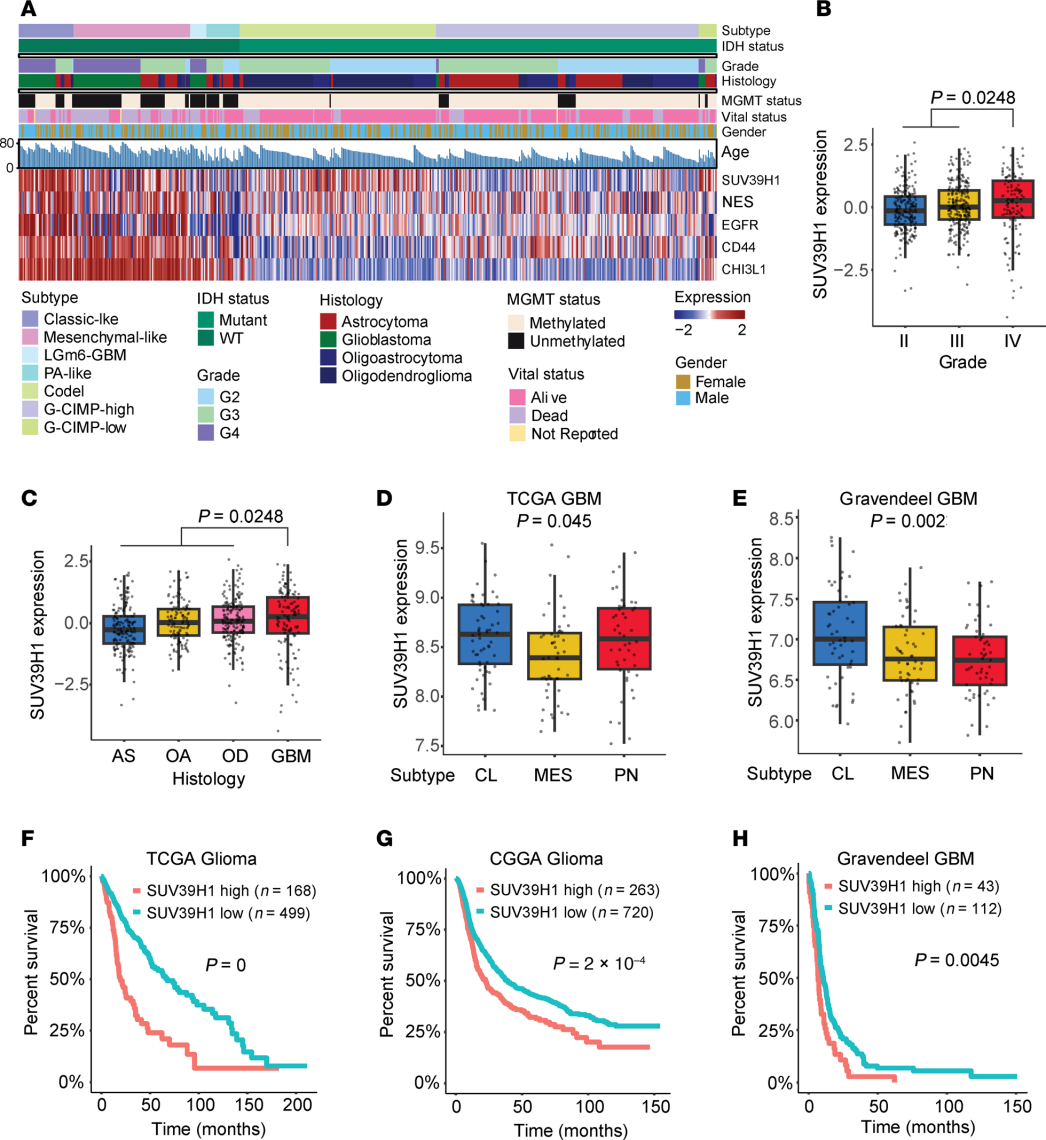
SUV39H1 serves as a therapeutic target and prognostic indicator in GBM. (**A**) RNA-seq, whole-exome-seq, and clinical phenotype data from TCGA GBM and low-grade glioma (LGG) datasets were integrated to visualize expression patterns of SUV39H1, NES, EGFR, and CD44 across glioma types. (**B**) SUV39H1 expression across glioma grades. A 2-sample Wilcoxon’s rank-sum test was used to compare SUV39H1 expression levels between grades II+III and grade IV. (**C**) SUV39H1 expression across glioma subtypes, including astrocytoma (AS), oligoastrocytoma (OA), oligodendroglioma (OD), and GBM. A 2-sample Wilcoxon’s rank-sum test was conducted to determine significant differences in expression levels, comparing the non-GBM group (AS, OA, OD) to the GBM group. The displayed *P* value reflects this comparison. (**D** and **E**) SUV39H1 expression in GBM subtypes in the TCGA (**D**) and Gravendeel (**E**) datasets. In the box-and-whisker plots, the boxes represent the interquartile range (IQR) from the first quartile (Q1) to the third quartile (Q3), with the horizontal line inside indicating the median. The whiskers extend to the smallest and largest values within 1.5 × IQR from Q1 and Q3, respectively, while outliers beyond this range are shown as individual dots. CL, classical; MES, mesenchymal; PN, proneural. Statistical analysis was conducted using the Kruskal-Wallis test, with *P* values indicating the significance of differences among the 3 groups. (**F**–**H**) Survival curves of patients with higher and lower SUV39H1 expression in TCGA glioma (**F**), CGGA glioma (**G**), and Gravendeel GBM (**H**) datasets. Kaplan-Meier survival analysis was used, with patients divided into high and low SUV39H1 expression groups based on the median expression level as a cutoff. Statistical significance between survival curves was determined using the log-rank test.

To explore the clinical importance of SUV39H1, we analyzed its expression across glioma samples in the TCGA dataset. SUV39H1 expression was positively correlated with the classic-like glioma subtype and markers such as NES and EGFR, with a lower correlation with mesenchymal-like markers like CD44 and CHI3L1 (Figure 9A). High SUV39H1 expression was seen in tumors with wild-type IDH and older patients (Figure 9A) and increased with tumor grade, peaking in grade IV gliomas, which include GBM (*P* = 0.0248; Figure 9B). GBM tumors exhibited the highest SUV39H1 expression (*P* = 0.0248; Figure 9C), particularly within the classical subtype compared with mesenchymal and proneural subtypes in both TCGA and Gravendeel datasets (Figure 9, D and E). Regarding prognosis, elevated SUV39H1 expression was linked to poorer outcomes across glioma grades in both the CGGA and TCGA datasets (Figure 9, F and G). While SUV39H1 was not prognostic in the TCGA GBM cohort (*P* = 0.4849; Supplemental Figure 6), high expression was associated with poorer prognosis in the Gravendeel GBM dataset (*P* = 0.0045; Figure 9H). These findings highlight SUV39H1 as a potentially valuable therapeutic target and prognostic indicator in glioma and GBM.

## Discussion

**Figure 10:**
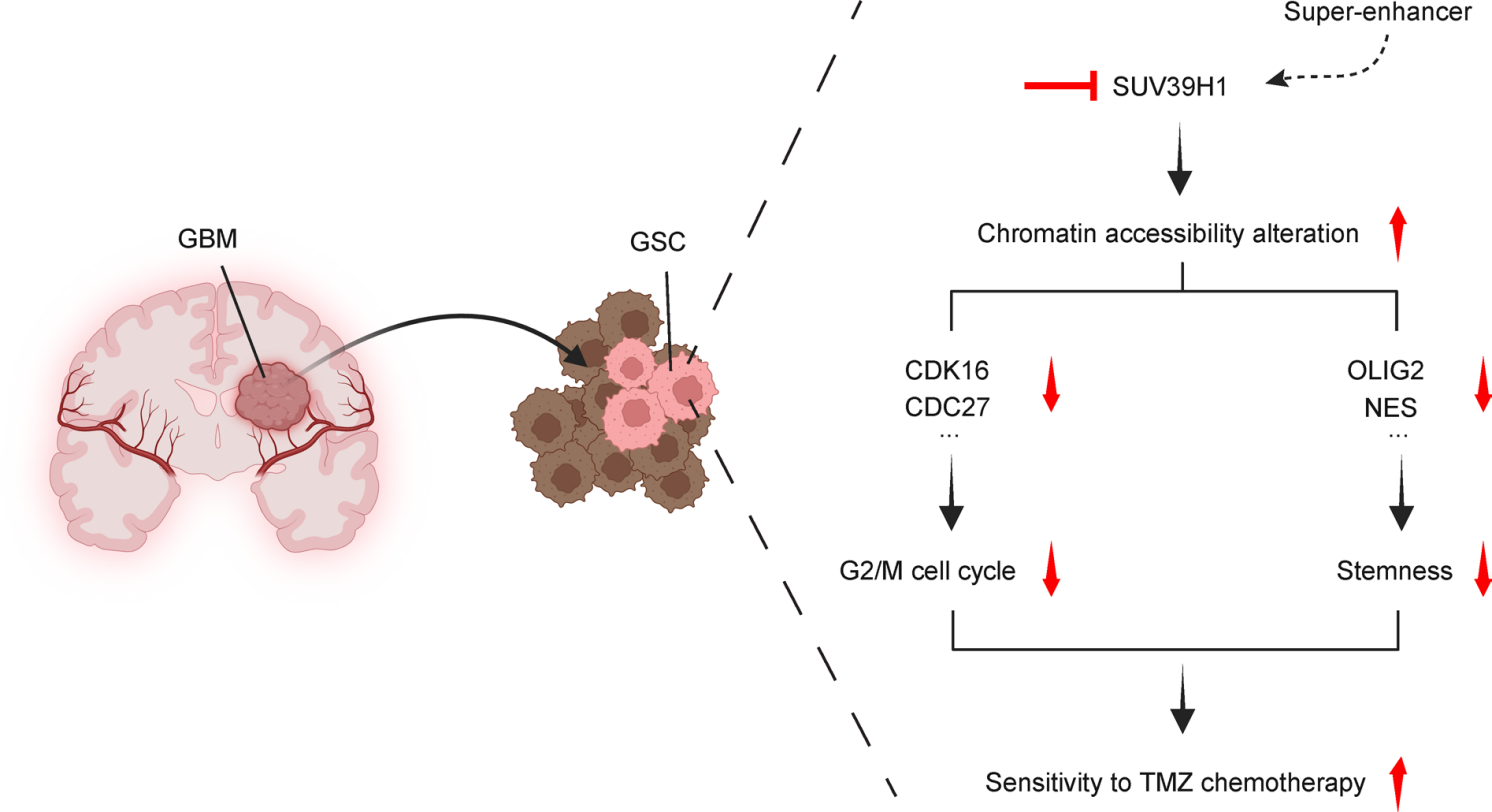
Schematic model of SUV39H1 targeting in GSCs. This working model illustrates the signaling cascade involving the super-enhancer, SUV39H1, chromatin accessibility, G_2_/M cell cycle regulation, stemness, and sensitivity to TMZ chemotherapy.

GSCs play a pivotal role in GBM tumor initiation, progression, and recurrence. Their inherent resistance to standard therapies, such as chemotherapy and radiation, presents a significant clinical challenge. To address this, extensive research has been directed toward identifying and targeting the genes and signaling pathways that regulate GSC stemness and survival, aiming to eliminate GSCs directly or sensitize them to conventional treatments. This study identifies SUV39H1 as a critical dependency in GSCs, regulating key pathways involved in cell cycle progression, stem cell maintenance, and cell death. Targeting SUV39H1 leads to reduced GSC maintenance, increased sensitivity to TMZ chemotherapy, and impaired GBM tumor growth in mice (Figure 10).

SUV39H1 plays a crucial role in the development and progression of various cancers. For instance, SUV39H1-mediated H3K9me3 can silence the tumor suppressor gene *p16INK4a*, promoting uncontrolled cell proliferation in acute myeloid leukemia and lung cancer cells (24, 25). SUV39H1 deposits H3K9me3 at the promoter of the cell death receptor–encoding *FAS* gene, repressing expression and inhibiting apoptosis in metastatic colorectal cancer cells (26). Our previous study revealed that SUV39H1 signaling contributes to the maintenance of repetitive elements and genomic stability across various cancer cell types (21). In this study, we investigated the functional importance of SUV39H1 in cancer stem cells in GBM. We found that, in addition to regulating cell proliferation, SUV39H1 is essential for GSC stemness maintenance through regulation of chromatin accessibility. Interestingly, a previous study in bladder cancer stem cells showed that SUV39H1 maintains the self-renewal of these cells by repressing the transcription factor GATA3, leading to the upregulation of STAT3 and maintaining stemness (27). More recently, another study linked SUV39H1 with cancer stemness in diffuse intrinsic pontine gliomas, reporting that targeting SUV39H1 led to the downregulation of growth factor receptor signaling and stemness-related programs, resulting in inhibited glioma cell growth and increased cell death (22). However, whether SUV39H1 also regulates stemness maintenance in cancer stem cells of other cancer types remains to be determined in future studies.

TMZ is a first-line chemotherapy agent for GBM that exerts its cytotoxic effects by methylating DNA at specific sites. This methylation creates mismatched base pairs, leading to DNA double-strand breaks (DSBs), disruption of the cell cycle at the G_2_/M checkpoint, and eventually, apoptosis (28–30). However, GSCs are resistant to TMZ treatment. In this study, we demonstrated that targeting SUV39H1 sensitized GSCs to TMZ treatment in vitro. This effect is likely mediated by several mechanisms linked to the role of SUV39H1 in maintaining GSC stemness and genome stability. Targeting SUV39H1 reduced GSC stemness, which is closely associated with DNA damage response and DNA repair efficiency (31–33), likely impairing the DSB repair process. Furthermore, our unpublished data indicate that targeting SUV39H1 upregulates IFN-β expression in GSCs. Increased IFN-β has been reported to suppress MGMT, a key DNA repair enzyme that mitigates TMZ-induced lesions (34–36). This suppression could further compromise DNA repair and enhance TMZ efficacy. Additionally, SUV39H1 targeting induces H3K9me3-mediated heterochromatin destabilization, as suggested by our unpublished findings, resulting in increased genome instability and DNA damage in GSCs. When combined with TMZ, the elevated DNA damage burden may overwhelm the repair mechanisms in GSCs, leading to enhanced mitotic catastrophe and cell death. Further experimental validation is essential to fully elucidate these mechanisms, assess their relative contributions, and confirm these effects in in vivo models.

Chaetocin, a natural metabolite derived from *Chaetomium* species, was initially identified in 2005 as an inhibitor of the histone lysine methyltransferase SUV39H1 (37). Subsequent research revealed that chaetocin also targets other proteins, such as thioredoxin reductase-1 (TrxR1), an important enzyme involved in cellular oxidative stress responses (38). By inhibiting TrxR1 and disrupting the TrxR/Trx pathway, chaetocin increases reactive oxygen species (ROS) levels, which activate death-receptor gene transcription and trigger receptor-mediated apoptosis in an ROS-dependent manner. Previous research revealed that chaetocin exerts a dual mechanism by directly inhibiting SUV39H1 and indirectly affecting it via ROS production in acute myeloid leukemia cells (39). In GSCs, chaetocin reduces proliferation and stemness, reflecting effects similar to those observed with SUV39H1 KD. However, chaetocin may also engage additional SUV39H1-independent mechanisms, such as ROS generation and other signaling pathways, which could further contribute to these observed effects. Further investigation will be essential to distinguish the specific pathways and mechanisms by which chaetocin exerts these additional impacts on proliferation and stemness. Chaetocin has been widely used as a proxy for SUV39H1 inhibition in numerous in vitro and in vivo preclinical studies across various cancer types (22, 40–45). However, no commercially available compound currently targets SUV39H1 with specificity. This underscores the need to develop selective SUV39H1 inhibitors that could offer a more precise therapeutic strategy, particularly for targeting GSCs in GBM treatment.

In this study, we utilized single-cell RNA-seq to map the transcriptomic landscape of GBM, identifying distinct cell populations and revealing the expression of SUV39H1 across various cell types. While the primary focus was on the role of SUV39H1 in GSCs, it is evident that SUV39H1 also regulates the functions of other cell types in the GBM microenvironment. Previous research showed in tumor-infiltrating cytotoxic T lymphocytes (CTLs) in human colon carcinoma that SUV39H1 mediated H3K9me3 marks at the promoters of CTL effector genes (e.g., *GZMB*, *PRF1*, *FASLG*, and *IFNG*). This enrichment repressed their expression and diminished CTL cytotoxicity, thereby facilitating tumor immune evasion. Targeting SUV39H1 increased the expression of these effectors in CTLs, resulting in suppression of colon tumor growth (46). Another study in diffuse large B cell lymphoma revealed that SUV39H1 upregulated CD86^+^ and CD163^+^ tumor–associated macrophages, suggesting a mechanism for lymphoma progression (47). Recent studies also underscore the role of SUV39H1 in regulating chimeric antigen receptor (CAR) T cells. Targeting SUV39H1 enhanced the persistence and antitumor effects of CAR T cells in lung and disseminated solid tumor models (48), as well as in leukemia and prostate cancer models (49). Understanding the roles of SUV39H1 in various cell types within the tumor microenvironment is crucial for evaluating the therapeutic potential of SUV39H1 inhibitors for GBM treatment. Ideally, these inhibitors should selectively target cancer cells and protumor cells, such as regulatory T cells and myeloid-derived suppressor cells, while sparing or even benefiting anti-tumor cells, such as CTLs and natural killer cells.

While SUV39H1 appears to be a promising therapeutic target for GBM, several challenges need to be considered for its clinical translation. First, the development of SUV39H1-specific inhibitors with favorable pharmacokinetic and pharmacodynamic profiles is critical. These inhibitors must effectively penetrate the blood-brain barrier to reach GSCs within the tumor microenvironment, which remains a significant hurdle for drug delivery in brain cancers. Strategies such as nanoparticle-based delivery systems may improve distribution (50–52). Second, GBM is a highly heterogeneous disease, with patient variability in tumor subtype, genetic mutations, and microenvironmental factors that can impact the effectiveness of SUV39H1 targeting. Stratifying patients based on molecular profiles, such as SUV39H1 expression levels or associated biomarkers, could optimize treatment responses and inform precision medicine approaches. Finally, safety concerns regarding off-target effects should be thoroughly addressed. While SUV39H1 inhibition selectively targets GSCs in vitro, preclinical validation in orthotopic GBM models is essential to assess potential effects on normal cells, ensuring minimal impact on healthy populations. This will be key to determining safe therapeutic windows and long-term tolerability.

In conclusion, our integrative approach, combining in vitro and in vivo experiments with bioinformatics analyses, provides compelling evidence that SUV39H1 is a key regulator of GSC functions. Targeting SUV39H1 could offer insights into strategies to improve outcomes for GBM patients.

## Methods

### Sex as a biological variable.

Our study examined male and female animals, and similar findings are reported for both sexes.

### Cell culture.

Lenti-X-293T cells (Takara Bio, 632180) and U118 cells were cultured in DMEM (Gibco, 11995065) supplemented with 10% FBS (Corning, 35015CV). Patient-derived GSC3565, GSC1914, GSC23, and GSC839 cells were provided by Jeremy Rich (UPMC Hillman Cancer Center, Pittsburgh, Pennsylvania, USA). The GSCs were cultured according to established protocols (53, 54). To minimize cell culture artifacts, patient-derived xenografts were produced and maintained as a renewable source of tumor cells.

### Lentivirus preparation and infection.

Lenti-X-293T cells cultured in DMEM were transfected with lentivirus packaging plasmids (pMD2.g and psPAX2), along with either control or SUV39H1 shRNA vectors (Sigma-Aldrich Mission shRNA, TRCN0000285355 [shSUV39H1-1] and TRCN0000275322 [shSUV39H1-2]), SOX2 shRNA (TRCN0000231642), OLIG2 shRNA (TRCN0000018158), or pHAGE PGK-GFP-IRES-LUC-W (Addgene, 46793) using jetPRIME transfection reagent (Polyplus, 101000001). After 24 hours, the DMEM was replaced with either GSC culture media (for GSC infection) or fresh DMEM (for NSGC and U118 infection). Cells were then incubated for an additional 48 hours at 37°C to produce lentiviral particles. The supernatant containing lentivirus was collected and stored at –80°C. For infection, GSCs, NSGCs, or U118 cells (4 × 10^5^ cells per well in a 6-well plate) were exposed to lentivirus for 18 hours, followed by a medium change and incubation for an additional 48 hours.

### Cell cycle analysis.

GSC3565, GSC1914, NSGC3565, and U118 cells transduced with shRNAs were washed with cold PBS, and fixed in cold 100% ethanol, and stored at –20°C for more than 24 hours. After washing with cold PBS, the cells were stained with FxCycle PI/RNase Staining Solution (Invitrogen, F10797) for 15 minutes at room temperature, following the manufacturer’s instructions. Cells were then analyzed using a CytoFlex LX flow cytometer (Beckman Coulter). Cell cycle data were analyzed using FlowJo software (BD Biosciences).

### Tumorsphere assay and ELDA.

For tumorsphere assays, GSCs expressing the indicated shRNAs were digested into single cells and seeded into 6-well plates (4 × 10^5^ per well for GSC3565, GSC23, and GSC839; 1 × 10^5^ per well for GSC1914). After 72 hours, the cells were imaged using an Axiovert 40 CFL inverted microscope (Zeiss), and tumorspheres were counted in selected fields. For chaetocin treatments, the GSCs were seeded into wells and treated with either vehicle or 50 nM chaetocin for 48 hours before imaging. ELDA is an assay that quantifies the frequency of self-renewing cells, essential for identifying cancer stem cells, and enhances accuracy by statistically analyzing sphere-forming ability. For ELDA, GSCs transduced with shRNAs were seeded into 96-well plates at varying densities in triplicate. After 13 days of incubation, wells containing tumorspheres were counted. Stem cell frequency was estimated using an online ELDA analysis tool (https://bioinf.wehi.edu.au/software/elda/).

### IHC.

Normal brain and GBM tissues (Supplemental Table 1) were obtained from the Enterprise Clinical Research Operations (ECRO) Biorepository at Indiana University Health Methodist Hospital with approval from the Institutional Review Board (IRB) for the collection of human biological materials. Tissues were processed in formalin-fixed, paraffin-embedded (FFPE) format, and the staining was conducted as previously described (55). The antibody used was anti-SUV39H1 (Thermo Fisher Scientific, PA5-29470; 1:100). For IHC staining of brain tissues extracted from NSG mice injected with control KD or SUV39H1-KD GSCs, the tissues were processed similarly to the above-described method. The antibodies used were anti-SUV39H1 (Thermo Fisher Scientific, PA5-29470; 1:100), Ki67 (Cell Signaling Technology, 9449; 1:1000), and OLIG2 (EMD Millipore, MABN50; 1:1000). All slides were imaged using an EasyScan Pro 6 scanner (Motic).

### Western blotting.

For Western blotting, GSCs were lysed in cold RIPA buffer (Thermo Fisher Scientific, 89901) supplemented with Protease and Phosphatase Inhibitor Mini Tablets (Thermo Fisher Scientific, A32961). The procedure was detailed previously (21). The following antibodies were used: anti-SUV39H1 rabbit antibody (Invitrogen, 702443; 1:1000), anti-OLIG2 rabbit antibody (Cell Signaling Technology, 65915; 1:1000), anti-GFAP mouse antibody (Cell Signaling Technology, 3670; 1:1000), anti–β-actin mouse antibody (Cell Signaling Technology, 3700; 1:1000), anti–α-tubulin rabbit antibody (Proteintech, 11224-1-AP; 1:4000), HRP-conjugated rabbit secondary antibodies (Invitrogen, 31460; 1:10,000), and mouse secondary antibodies (Invitrogen, 31430; 1:10,000).

### qPCR.

Total RNA was isolated using the RNeasy Plus Mini Kit (QIAGEN, 74136). cDNA synthesis was performed with the qScript cDNA SuperMix (Quantabio, 95048). qPCR reactions were carried out using PowerUp SYBR Green Master Mix (Applied Biosystems, 25742) in a CFX Duet Real-Time PCR System (Bio-Rad). The thermal cycling conditions were as follows: an initial denaturation at 95°C for 30 seconds, followed by 40 cycles of 95°C for 3 seconds, and 60°C for 20 seconds. Analyses were performed in triplicate for each data point. qPCR primers used for gene expression analysis are listed in Supplemental Table 2.

### Immunofluorescence and EdU assays.

For immunofluorescent staining of GSCs, coverslips (neuVitro, GG-12-laminin) were placed in 24-well plates and coated with matrigel (Corning, 356231). GSCs grown on the coverslips were washed with cold PBS, fixed with 4% paraformaldehyde, permeabilized with 0.1% Triton X-100 in PBS, and incubated in blocking buffer (1% BSA in PBS). Anti-OLIG2 rabbit antibody (Cell Signaling Technology, 65915; 1:200) and Alexa Fluor 488–labeled anti-rabbit secondary antibody (Invitrogen, A11008; 1:500) were used. For costaining of SUV39H1 with OLIG2 and SOX2 in GBM tissues (Supplemental Table 3), the slides were simultaneously stained with primary antibodies anti-SUV39H1 (Thermo Fisher Scientific, PA5-29470; 1:500), anti-OLIG2 (EMD Millipore, MABN50; 1:1000), and anti-SOX2 (EMD Millipore, MABN4343; 1:500). Alexa Fluor 488–labeled anti-rabbit secondary antibody (Invitrogen, A11008; 1:500) and Alexa Fluor 594–labeled anti-mouse secondary antibody (Invitrogen, A11005; 1:500) were used. For the EdU assay, GSCs grown on matrigel-coated coverslips were labeled with EdU for 4 hours, and the subsequent steps were performed according to the instructions of the Click-iT EdU Cell Proliferation Kit (Invitrogen, C10337). All images were captured using a Nikon DS-Fi3 fluorescence microscope.

### Cell proliferation and viability assays.

The cells were seeded in 96-well plates (2 × 10^3^ cells per well), and relative cell numbers were measured on specified days using the CellTiter-Glo kit (Promega, 9863) following the manufacturer’s instructions. For the in vitro drug treatment, GSC3565 cells in 96-well plates were treated with varying concentrations of TMZ (0, 200, 400, 800 nM) and chaetocin (0, 10, 20, 40 nM), either individually or in combination. After 72 hours, cell viability was measured using the CellTiter-Glo kit. For GSC1914 cells, the concentrations used were TMZ (0, 750, 1000, 1500 nM) and chaetocin (0, 20, 40, 80 nM). Synergy scores were determined using the SynergyFinder web tool (https://synergyfinder.org/). All experiments were conducted in triplicate.

### In vivo xenograft studies.

NSG mice (male and female, aged 4 to 6 weeks; Animal Facility at Sanford Burnham Prebys Medical Discovery Institute) were intracranially injected with either control KD or SUV39H1-KD GSC3565-luc cells (expressing luciferase; 5 × 10^4^ cells per mouse). Tumor size was monitored via luciferase signal using a IVIS Spectrum Imager (Xenogen). Animals were sacrificed if weight loss exceeded 20% and/or they displayed neurological signs, including but not limited to lethargy, ataxia, or seizures. On day 29, the brains were extracted, and hematoxylin and eosin (H&E) staining was performed on sections for histological analysis. IHC staining of SUV39H1, OLIG2, and Ki67 was also performed.

### RNA-seq.

Total RNA isolated from GSC3565 and GSC1914 cells with control KD or SUV39H1 KD was subjected to RNA-seq at Novogene Corporation. Quality control of the raw RNA-seq reads was performed using FastQC (https://github.com/s-andrews/FastQC) and fastp (https://github.com/OpenGene/fastp), and adapter sequences were removed with TrimGalore (https://github.com/FelixKrueger/TrimGalore). Trimmed reads were aligned to the human reference genome (hg19) using STAR aligner (https://github.com/alexdobin/STAR). Quantification of transcript abundance was performed using Salmon (https://github.com/COMBINE-lab/salmon) in quasi-mapping mode and the resulting quantification files were imported into R (https://www.r-project.org/) for differential expression analysis using the DESeq2 package. Differentially expressed genes (DEGs) were identified with a cutoff of log_2_(fold change) greater than 1 or less than –1 and an adjusted *P* value of less than 0.05. DEGs were visualized using volcano plots generated with the ggplot2 package. GSEA was performed using the preranked list of DEGs based on the log_2_(fold change) and adjusted *P* value. The GSEA visualization and enrichment were conducted using the R package clusterProfiler. Enriched pathways and gene ontology (GO) terms were represented as bubble plots using Cytoscape (https://cytoscape.org/).

### ATAC-seq.

GSC3565 cells treated with vehicle or 50 nM chaetocin for 48 hours were subjected to ATAC-seq at the Center for Medical Genomics at Indiana University School of Medicine. ATAC-seq libraries were prepared following the Omni-ATAC protocol. Briefly, GSCs were washed with cold PBS and lysed in ATAC lysis buffer (10 mM Tris-HCl, pH 7.4, 10 mM NaCl, 3 mM MgCl_2_, 0.1% Igepal CA-630). Nuclei were collected by centrifugation and resuspended in transposition reaction mix containing Tn5 transposase (Illumina, FC-121-1030) and incubated at 37°C for 30 minutes. The transposed DNA was purified using the DNA Clean & Concentrator-5 (Zymo, D4003) and PCR was amplified using Nextera PCR primers. The amplified libraries were purified with the KAPA HiFi HotStart ReadyMix (Roche, KK2602) and quantified using a Qubit fluorometer (Thermo Fisher Scientific). Libraries were sequenced on an Illumina platform to generate paired-end reads. Sequencing reads were trimmed for adapter sequences using Trim Galore and aligned to the human reference genome (hg19) using Bowtie2 (https://bowtie-bio.sourceforge.net/bowtie2/index.shtml). Aligned reads were filtered to remove duplicate reads, mitochondrial reads, and low-quality reads using SAMtools (https://github.com/samtools/samtools) and sambamba (https://lomereiter.github.io/sambamba/). Peaks were called using MACS2 (https://hbctraining.github.io/Intro-to-ChIPseq/lessons/05_peak_calling_macs.html) to identify regions of open chromatin. The resulting peak files were further processed to generate a high-confidence set of peaks by filtering out blacklist regions and peaks overlapping with ENCODE blacklisted regions. The number of reads within each peak was quantified using featureCounts from the Subread package. Differentially accessible regions between conditions were identified using DESeq2, with an adjusted *P* value of less than 0.05 considered significant. Peaks were annotated to the nearest genes using HOMER (http://homer.ucsd.edu/homer/motif/), and GO enrichment analysis was performed on the annotated genes using the clusterProfiler package. Heatmaps and average signal profiles of ATAC-seq data were generated using DeepTools (https://deeptools.readthedocs.io/en/latest/). The bigWig files for visualization were created using bedGraphToBigWig (https://www.encodeproject.org/software/bedgraphtobigwig/). The Integrative Genomics Viewer (https://igv.org/) was used to visualize the ATAC-seq signal tracks across the genome.

To integrate ATAC-seq data with RNA-seq data, the overlap of differentially accessible regions with DEGs was analyzed using BEDTools intersect (https://bedtools.readthedocs.io/en/latest/content/tools/intersect.html). The genomic regions enrichment of annotations tool (GREAT) was used to identify the biological relevance of the genomic regions identified.

### Single-cell RNA-seq analysis.

Single-cell RNA-seq data were downloaded from the CELLxGENE database (https://cellxgene.cziscience.com/ Accessed May 21, 2024.) and integrated and analyzed using Seurat (v4.4.0; https://satijalab.org/seurat/). Prior to integration, quality control was performed to remove low-quality cells (<200 genes detected, >10% mitochondrial reads) and potential doublets. Following quality control, cells were annotated based on the cell type information provided in the CELLxGENE database. Data integration was achieved using Seurat’s SCTransform workflow, followed by batch effect correction with the Harmony algorithm. Dimensionality reduction was performed using Principal Component Analysis (PCA) on the top 2000 variable genes, with the first 30 principal components used for downstream analysis. Cells were clustered based on their gene expression profiles using the Louvain algorithm with a resolution of 0.8. UMAP was used for visualization of the high-dimension data in 2 dimensions. Cell type annotations were refined using canonical markers, with a comprehensive list of markers used for annotation provided in Supplemental Table 4. Differential expression analysis was performed using Wilcoxon’s rank-sum test, and significant genes were identified based on an adjusted *P* value of less than 0.05 and log_2_(fold change) of greater than 1, with Benjamini-Hochberg correction for multiple testing. Specific markers for GSCs (including OLIG2, SOX2, NES) and other cell types were visualized on UMAP plots using a color gradient to represent expression intensity.

### Statistics.

All data are presented as mean ± SD. A *P* value of less than 0.05 was considered significant. The statistical methods for each experiment are detailed in the figure legends.

### Study approval.

All animal experiments were performed under an animal protocol approved by the Institutional Animal Care and Use Committee (IACUC) at Sanford Burnham Prebys Medical Discovery Institute. This work did not contain research involving humans.

### Data availability.

Raw data for all graphs are reported in the Supporting Data Values file. The RNA-seq data were deposited in the NCBI GEO database under accession number GSE273012. The ATAC-seq data are also available in the GEO database under accession number GSE273013. Other data described in this article are available upon request from the corresponding author.

## Author contributions

CL, QX, SG, BC, YD, GVV, and JS performed the experiments and analyzed the results. TYH, CS, RLC, YAW, QRL, and KPN provided resources. JS developed the methodology and drafted the manuscript.

## Supplementary Material

Supplemental data

Unedited blot and gel images

Supporting data values

## Version 1 03/10/2025

Electronic publication
