# Supplementary material for: SUV39H1 maintains cancer stem cell chromatin state and properties in glioblastoma
Source: JCI Insight. 2025 Mar 10;10(5):e186344. doi: 10.1172/jci.insight.186344 (PMC11949068; doi:10.1172/jci.insight.186344)
Supplement: Supplemental data [file jciinsight-10-186344-s286.pdf]

Supplementary Table 1. Information for 4 normal and 9 GBM tissues for IHC

Patient ID	Project Sample ID	Cell Type	Age at Collection	Gender/Sex	Race
PID-118597	3007QBV	Non-Neoplastic	53y	Female	White
PID-132099	3004WMW	Non-Neoplastic	28y	Female	White
PID-144834	3012WWC	Non-Neoplastic	30y	Female	White
PID-144940	3012RJP	Non-Neoplastic	71y	Male	White
PID-118597	3007QKU	Glioblastoma	53y	Female	White
PID-129177	3003XXC	Glioblastoma	70y	Male	White
PID-133071	3008QHT	Glioblastoma	57y	Male	White
PID-133071	3009ABW	Glioblastoma	58y	Male	White
PID-134067	3004PBW	Glioblastoma	59y	Female	White
PID-135231	3009AJK	Glioblastoma	56y	Female	White
PID-142279	3009RGF	Glioblastoma	35y	Male	Hispanic/Latino
PID-136344	3009FPN	Glioblastoma	62y	Male	White
PID-135323	3012BVD	Glioblastoma	64y	Female	White

Supplementary Table 2. qPCR primers

Gene name	Forward Primer	Reverse Primer
SUV39H1	CCTGCCCTCGGTATCTCTAAG	ATATCCACGCCATTTCACCAG
OLIG2	TGGCTTCAAGTCATCCTCGTC	ATGGCGATGTTGAGGTCGTG
MYC	AAAGGCCCCCAAGGTAGTTA	GCACAAGAGTTCCGTAGCTG
GFAP	CTGGAGAGGAAGATTGAGTCGC	ACGTCAAGCTCCACATGGACCT
ACTIN	CATGTACGTTGCTATCCAGGC	CTCCTTAATGTCACGCACGAT
CDK16	TCCGTCGTGTCAGCCTATCT	TCATGTTCCAGTCTGATCTCCTT
CUL3	TGTGGAGAACGTCTACAATTTGG	GCGCCTCTGTCTACGACTT
SLC7A11	TCTCCAAAGGAGGTTACCTGC	AGACTCCCCTCAGTAAAGTGAC
NES	CAGCGTTGGAACAGAGGTTGG	TGGCACAGGTGTCTCAAGGGTAG
GPX4	GAGGCAAGACCGAAGTAAACTAC	CCGAACTGGTTACACGGGAA
FTH1	CCCCCATTTGTGTGACTTCAT	GCCCGAGGCTTAGCTTTCATT
IRF1	ATGCCCATCACTCGGATGC	CCCTGCTTTGTATCGGCCTG
IL1B	ATGATGGCTTATTACAGTGGCAA	GTCGGAGATTCGTAGCTGGA
HMOX1	AAGACTGCGTTCCTGCTCAAC	AAAGCCCTACAGCAACTGTCG
SOX2	TCCCGTATGAAAGCATCGTGG	CCCATTTGGGTAGATCAGGTAAC
OLIG2	TGGCTTCAAGTCATCCTCGTC	ATGGCGATGTTGAGGTCGTG

Supplementary Table 3. Information for 3 GBM tissues for immunofluorescence staining

Patient ID	Project Sample ID	Cell Type	Age at Collection	Gender/Sex	Race
PID-135231	3009AJK	Glioblastoma	56y	Female	White
PID-132241	3007TXH	Glioblastoma	68y	Male	White
PID-142653	3009PXK	Glioblastoma	68y	Male	White

Supplementary Table 4. List of markers used for cell type annotations

Cell Type	Marker Genes
Monocyte	LYZ, S100A8, S100A9, CXCL8, FTH1, SAT1, APOE, SPP1
Neutrophil	S100A8, S100A9, CXCL8
Macrophage	LYZ, S100A8, S100A9, FTH1, SAT1, APOE, SPP1
Microglial cell	HLA-DRA, HLA-DQB1, SPP1
Mature T cell	CD3D, CD3E, CD7
Natural killer cell	NKG7, CTSW, CD79A, CD79B, RPL13A, RPLP2
B cell	CD79A, CD79B, HLA-DRA, HLA-DQB1
Plasma cell	MZB1
Dendritic cell	HLA-DRA, HLA-DQB1, CPA3, VWA5A, TPSAB1, PLP1, SPP1
Mast cell	CPA3, TPSAB1
Oligodendrocyte	PLP1, MBP
Astrocyte	AQP4, GPM6A
Malignant cell	VWA5A, SPP1
Neuron	GPM6A, NOVA1, SYT1, RBFOX1, FGFR3
Oligodendrocyte precursor cell	PDGFRA, CD74, MBP
Radial glial cell	S100A4, FTL
Endothelial cell	SLC44A1, SPARCL1
Mural cell	S100A4, SPP1

SUV39H1 Maintains Cancer Stem Cell Chromatin State and Properties in Glioblastoma

Chunying Li, Qiqi Xie, Sugata Ghosh, Bihui Cao, Yuanning Du, Giau Vo, Timothy Y. Huang, Charles Spruck, Richard L. Carpenter, Y. Alan Wang, Q. Richard Lu, Kenneth P. Nephew, Jia Shen

Supplemental material

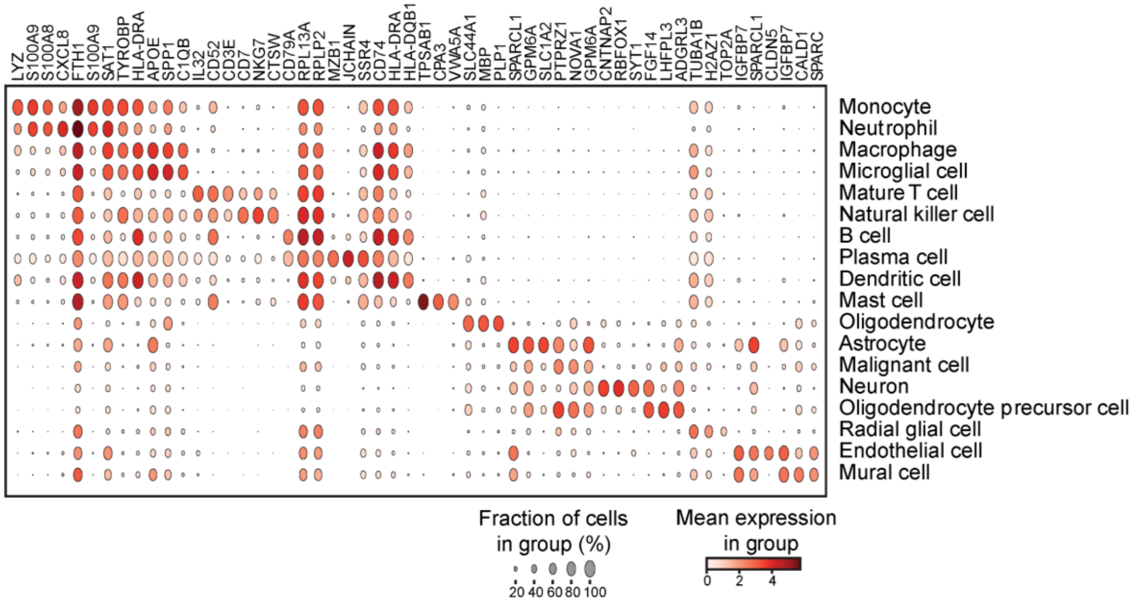

Supplemental Figure 1. Cell type-specific markers and their expression in GBM. Dot plot showing the expression of cell type-specific markers across different cell populations identified in the single-cell RNA-seq data. The size of each dot represents the fraction of cells expressing the marker in each cell type, and the color intensity represents the mean expression level within the group.

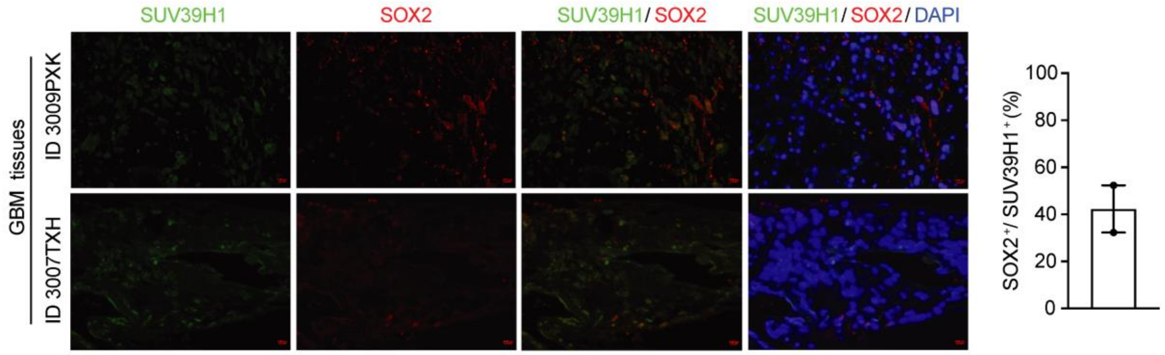

Supplemental Figure 2. Immunofluorescence staining of SUV39H1 and SOX2 in GBM tissues. Representative images (left panel) and quantification (right panel) of immunofluorescence staining showing co-localization of SUV39H1 (green) and SOX2 (red) in GBM tissues (n=2).

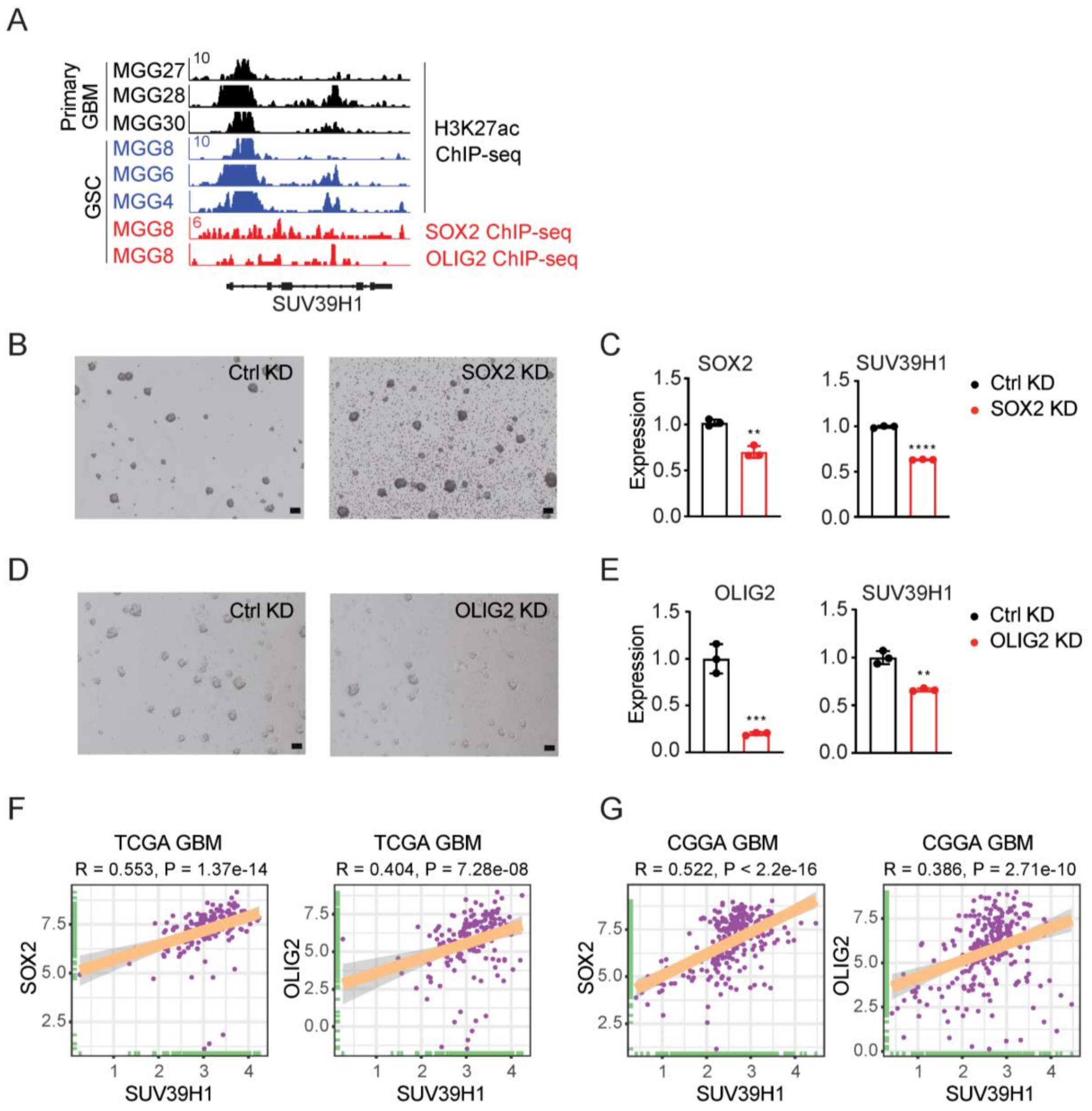

Supplemental Figure 3. SUV39H1 expression is regulated by super-enhancer in GSCs. (**A**) H3K27ac ChIP-seq signal tracks display the *SUV39H1* locus in specified primary GBM samples and GSCs, with SOX2 and OLIG2 ChIP-seq signals at the *SUV39H1* locus shown for MGG8 cells. (**B**-**E**) Representative images (**B**, **D**) and qPCR data (**C**, **E**) for GSC3565 with control or targeted gene KD. Unpaired t test. (**F**, **G**) Expression correlation analysis of SUV39H1, SOX2, and OLIG2 in the TCGA (**F**) and CGGA (**G**) GBM datasets. The Spearman correlation coefficient (R) was calculated to assess the strength and direction of the association, while the P value reflects the statistical significance of the correlation. Data represent mean ± SD. $^{**}P < 0.01$, $^{***}P < 0.001$, $^{****}P < 0.0001$.

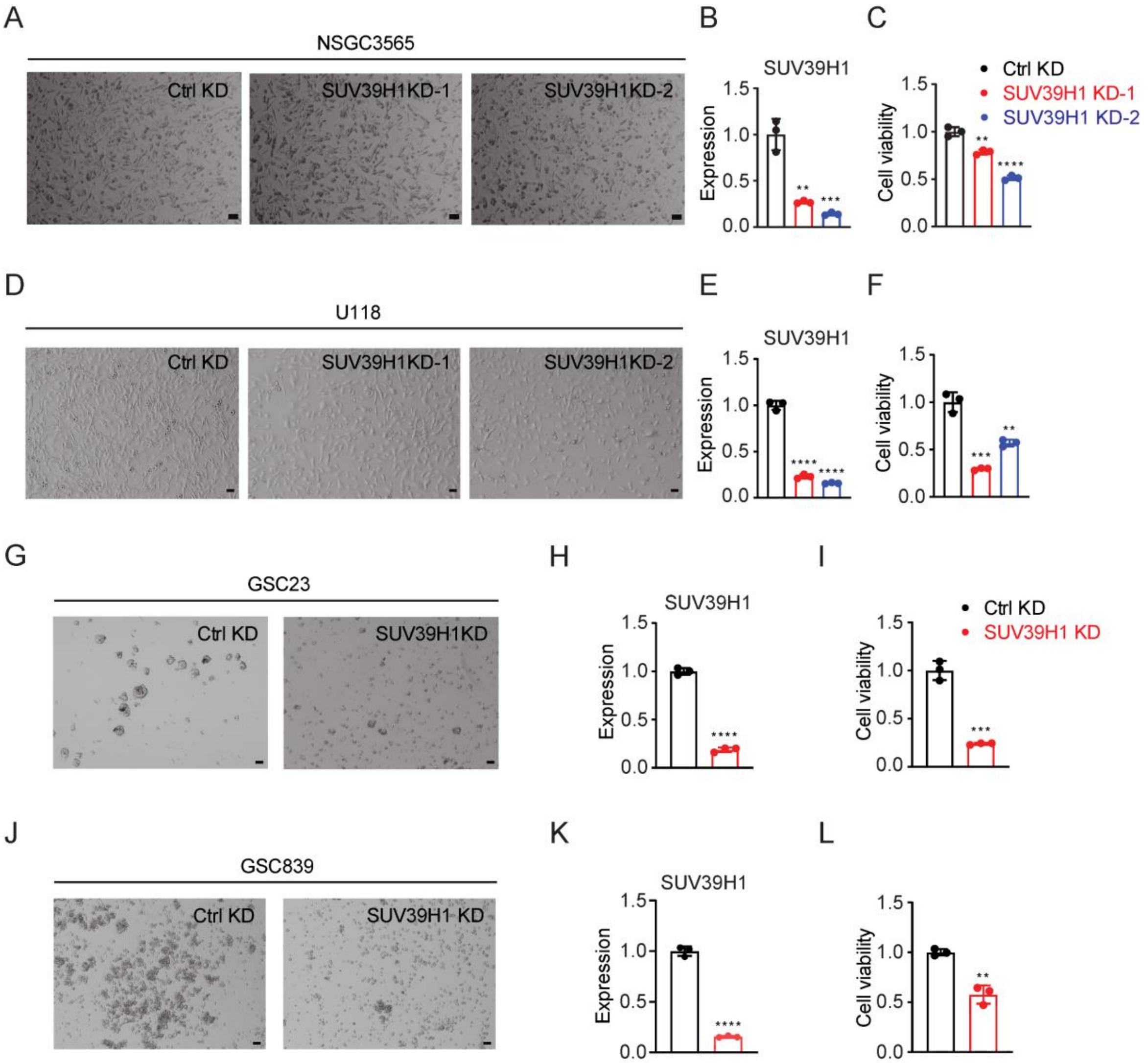

Supplemental Figure 4. Effects of SUV39H1 targeting in NSGCs, U118, GSC23, and GSC839. (**A**-**L**) Representative images (**A**, **D**, **G**, **J**), qPCR data (**B**, **E**, **H**, **K**), and cell viability data (**C**, **F**, **I**, **L**) for the indicated cells with control or SUV39H1 KD. Unpaired t test. Data represent mean ± SD. **$P < 0.01$, ***$P < 0.001$, ****$P < 0.0001$.

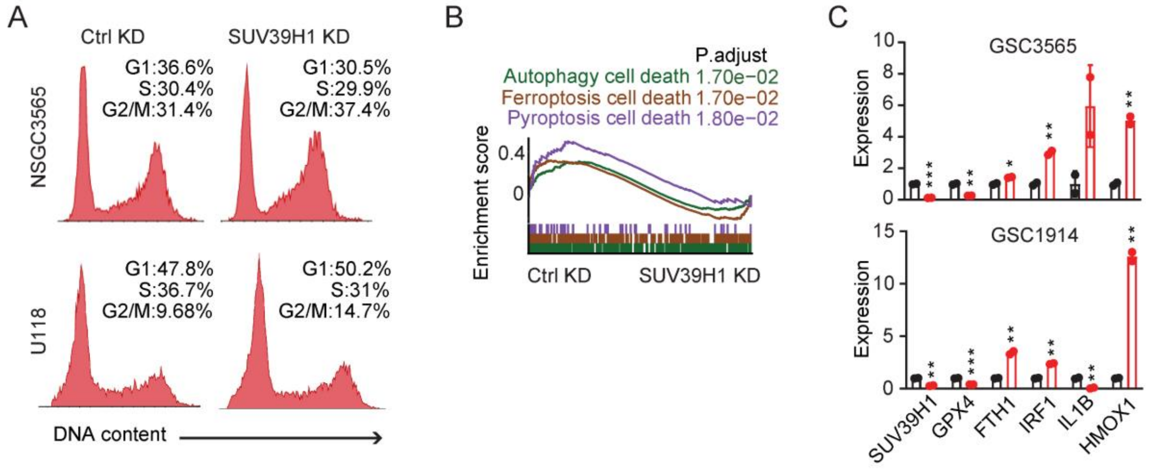

Supplemental Figure 5. Pathways regulated by SUV39H1. (**A**) Flow cytometry data showing cell cycle alteration in NSGCs and U118 cells with SUV39H1 KD. (**B**) GSEA plot showing enrichment of cell death pathways in SUV39H1 KD GSCs. (**C**) qPCR detection of cell death-related genes in GSC3565 and GSC1914 cells. Unpaired t test. Data represent mean ± SD. *P < 0.05, **P < 0.01, ***P < 0.001.

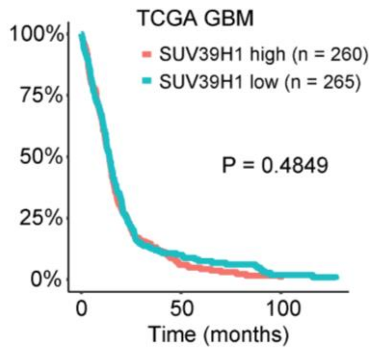

Supplemental Figure 6. Prognostic analysis of SUV39H1 expression in GBM. Survival curves for patients with high and low SUV39H1 expression in the TCGA GBM dataset. Kaplan-Meier survival analysis was used to assess the prognostic value of SUV39H1 expression, with patients divided into high and low expression groups based on the median expression level as the cutoff. Statistical significance between survival curves was determined using the log-rank test.
