# Supplementary material for: SUV39H1 maintains cancer stem cell chromatin state and properties in glioblastoma
Source: JCI Insight. 2025 Mar 10;10(5):e186344. doi: 10.1172/jci.insight.186344 (PMC11949068; doi:10.1172/jci.insight.186344)
Supplement: Unedited blot and gel images [file jciinsight-10-186344-s287.pdf]

Full unedited gel for Figure 3D

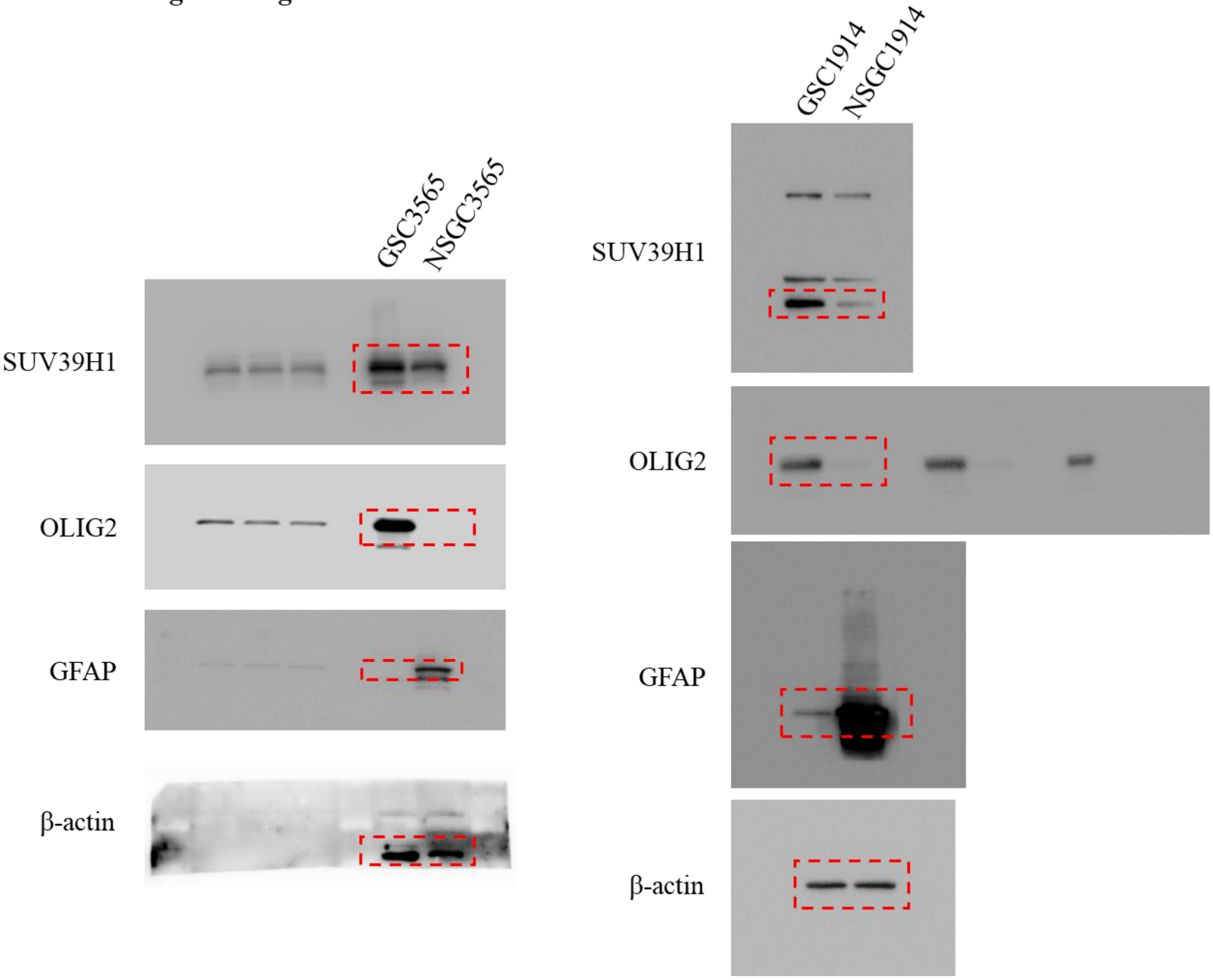

SUV39H1 rabbit antibody (Invitrogen, cat# 702443, 1:1000), OLIG2 rabbit antibody (Cell Signaling Technology, cat# 65915, 1:1000), GFAP mouse antibody (Cell Signaling Technology, cat# 3670, 1:1000), β-actin mouse antibody (Cell Signaling Technology, cat# 3700, 1:1000).

Full unedited gel for Figure 4A

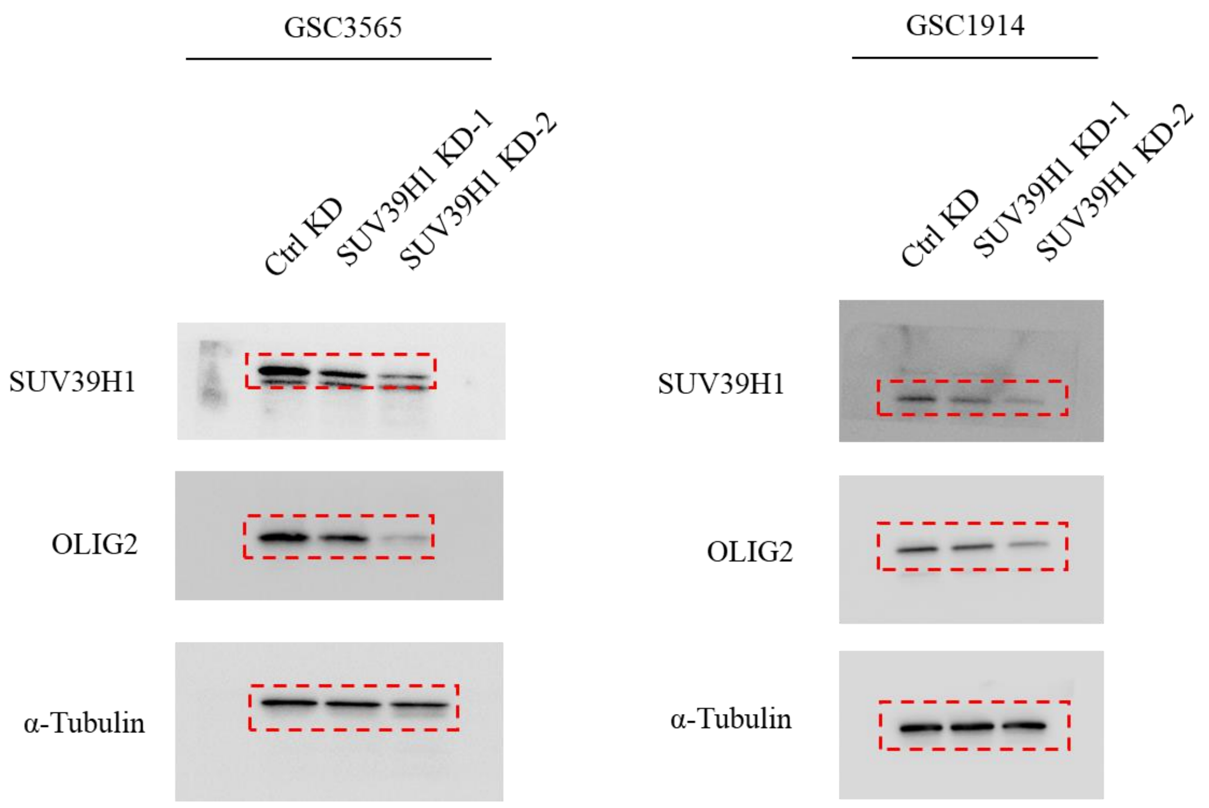

α-Tubulin rabbit antibody (Proteintech, cat# 11224-1-AP, 1:4000), SUV39H1 rabbit antibody (Invitrogen, cat# 702443, 1:1000), OLIG2 rabbit antibody (Cell Signaling Technology, cat# 65915, 1:1000).
